# HIV-1 Nef Targets MHC-I and CD4 for Degradation Via a Final Common β-COP–Dependent Pathway in T Cells

**DOI:** 10.1371/journal.ppat.1000131

**Published:** 2008-08-22

**Authors:** Malinda R. Schaefer, Elizabeth R. Wonderlich, Jeremiah F. Roeth, Jolie A. Leonard, Kathleen L. Collins

**Affiliations:** 1 Graduate Program in Immunology, University of Michigan, Ann Arbor, Michigan, United States of America; 2 Graduate Program in Cellular and Molecular Biology, University of Michigan, Ann Arbor, Michigan, United States of America; 3 Department of Microbiology and Immunology, University of Michigan, Ann Arbor, Michigan, United States of America; 4 Department of Internal Medicine, University of Michigan, Ann Arbor, Michigan, United States of America; Northwestern University, United States of America

## Abstract

To facilitate viral infection and spread, HIV-1 Nef disrupts the surface expression of the viral receptor (CD4) and molecules capable of presenting HIV antigens to the immune system (MHC-I). To accomplish this, Nef binds to the cytoplasmic tails of both molecules and then, by mechanisms that are not well understood, disrupts the trafficking of each molecule in different ways. Specifically, Nef promotes CD4 internalization after it has been transported to the cell surface, whereas Nef uses the clathrin adaptor, AP-1, to disrupt normal transport of MHC-I from the TGN to the cell surface. Despite these differences in initial intracellular trafficking, we demonstrate that MHC-I and CD4 are ultimately found in the same Rab7^+^ vesicles and are both targeted for degradation via the activity of the Nef-interacting protein, β-COP. Moreover, we demonstrate that Nef contains two separable β-COP binding sites. One site, an arginine (RXR) motif in the N-terminal α helical domain of Nef, is necessary for maximal MHC-I degradation. The second site, composed of a di-acidic motif located in the C-terminal loop domain of Nef, is needed for efficient CD4 degradation. The requirement for redundant motifs with distinct roles supports a model in which Nef exists in multiple conformational states that allow access to different motifs, depending upon which cellular target is bound by Nef.

## Author Summary

HIV is unique among viral pathogens in its capacity to cause chronic and progressive disease in almost all infected people. To accomplish this, HIV must evade the host immune response, especially cytotoxic T lymphocytes (CTLs), which normally function to lyse virally infected cells. HIV encodes a factor, Nef, which protects HIV infected cells from lysis by anti-HIV CTLs. To prevent CTL lysis, Nef interferes with the expression of host MHC-I, which is needed for CTL recognition of infected targets. A clear understanding of how Nef works has been hampered by its many complex functions. In addition to MHC-I, Nef protein disrupts the expression of multiple other cellular targets using different mechanisms and it is unclear how one protein can accomplish all these tasks. Here, we provide evidence that Nef acts as a highly flexible adaptor protein that is capable of utilizing different protein binding domains depending on which cellular target it is bound to. For example, we present evidence that Nef binding to MHC-I creates novel motifs that result in the recruitment of AP-1 and subsequently β-COP. This series of events results in the mis-localization of MHC-I from the cell surface to cellular degradative compartments, where MHC-I is destroyed.

## Introduction

The HIV-1 accessory protein, Nef, affects the biology of the infected cell in several ways to achieve conditions optimal for viral replication and spread. Nef alters the intracellular trafficking of important immune molecules, such as class I and II major histocompatibility complex proteins (MHC-I and MHC-II), CD4, CD28, and DC-SIGN [1]–[5]. Nef-dependent reduction of surface MHC-I protects HIV-infected primary T cells from recognition and killing by HIV-specific cytotoxic T lymphocytes (CTLs) *in vitro* [6]. Moreover, disruption of MHC-I expression by HIV-1 and SIV Nef provides a selective advantage under immune pressure *in vivo* [7]–[10]. CD4 downregulation by Nef is also essential for efficient viral spread. The rapid removal of CD4 prevents viral superinfection [11], and enables optimal viral particle production by eliminating detrimental CD4/HIV envelope interactions in the infected cell [12],[13].

Mutagenesis of protein-protein interaction domains has revealed that Nef uses genetically separable mechanisms to affect MHC-I and CD4 transport. Specifically, disruption of MHC-I surface expression requires an N-terminal α helix, a polyproline repeat, and an acidic domain in Nef [14],[15], while CD4 downregulation requires an intact dileucine motif, two diacidic motifs, and a hydrophobic pocket in Nef [15]–[18]. Amino acids necessary for the myristoylation [19],[20] and oligomerization [21] of Nef are required for the disruption of both MHC-I and CD4 surface expression.

Nef has the capacity to affect MHC-I transport at multiple subcellular locations; Nef blocks the export of newly-synthesized MHC-I from the secretory pathway and Nef expression results in a small increase in the rate of MHC-I internalization [22]. To accomplish this, Nef directly binds to the cytoplasmic tail of MHC-I early in the secretory pathway [23]–[26]. The Nef-MHC-I complex then actively recruits the clathrin adaptor protein complex AP-1, which targets MHC-I from the TGN to the endo-lysosomal network where it is ultimately degraded [25]. Recruitment of AP-1 primarily requires a methionine at position 20 in the N-terminal α helical domain of Nef and a tyrosine residue in the cytoplasmic tail of MHC-I. Additionally, the acidic and polyproline domains of Nef have recently been shown to stabilize this interaction [27],[28]. The normal function of AP-1 is to target proteins into the endosomal pathway and then recycle them back to the TGN. Thus, the AP-1 interaction with the Nef/MHC-I complex explains the targeting of MHC-I containing vesicles to the endosomal pathway and to the TGN. However, it does not explain accelerated degradation of MHC-I, hence other cellular factors may be involved [25].

The mechanism of Nef-induced CD4 internalization and degradation has been derived, in part, from correlating Nef function with the requirement for domains in the C-terminal flexible loop region of Nef that bind to cellular factors. The Nef dileucine motif (ExxxLL_165_) is needed for CD4 internalization and it binds to adaptor protein complexes AP-1, AP-2, and AP-3 [16], [29]–[36]. In addition, a diacidic motif, which is also required, enhances the interaction of Nef with AP-2 [37]. There is separate evidence that this diacidic motif may recruit the H subunit of the vacuolar ATPase (V1H) [38] to promote AP-2 recruitment [39]. Because the normal role of AP-2 is to link cargo to clathrin and promote internalization, it makes sense that this molecule would be necessary and indeed, the involvement of AP-2 has now been confirmed using RNAi knockdown in a number of cell systems [40]–[42].

After CD4 is internalized, it is targeted to lysosomes for degradation. There is evidence that this step requires β-COP [18], a component of COP-1 coats implicated in endosomal trafficking as well as transport through the early secretory pathway [43]–[45]. Specifically, there are defects in the Nef-dependent transport of CD4 into acidified vesicles at the non-permissive temperature in cells harboring a temperature sensitive ε-COP mutant [18]. Nef directly interacts with β-COP [46], and a second diacidic motif in the C-terminal loop domain of Nef has been demonstrated to mediate this interaction [18],[47], although, this result has not been reproducible by another group [48].

To more clearly understand the mechanism of altered MHC-I and CD4 trafficking observed in Nef-expressing cells, we directly compared these two processes in T cells that expressed Nef. We confirmed that Nef primarily affected MHC-I and CD4 at different subcellular locations and we demonstrated that the cytoplasmic tails of the respective molecules dictated which pathway was utilized. Despite the differences in initial trafficking, we found that HLA-A2 and CD4 co-localized in a discrete subset of vesicular structures. Upon further inspection, we determined that these structures also contained markers of late endosomes (Rab7) and to a lesser extent, the lysosomal marker, LAMP-1. Electron microscopy (EM) revealed that CD4 and HLA-A2 were found within MVBs of Nef-expressing T cells. HLA-A2 (but not CD4) was also found in tubulovesicular structures adjacent to the Golgi. In Nef expressing cells, reduction of β-COP expression reduced the targeting of HLA-A2 from the TGN to LAMP-1^+^ compartments and stabilized CD4 expression within endosomal compartments. Finally, we identified two separate domains within Nef that were necessary for these activities and for β-COP binding. These data support a model in which both MHC-I and CD4 are ultimately targeted to the lysosomes in Nef expressing cells by a final common pathway.

## Results

### The cytoplasmic tail dictates the pathway utilized by Nef to eliminate MHC-I and CD4 surface expression

**Figure 1:**
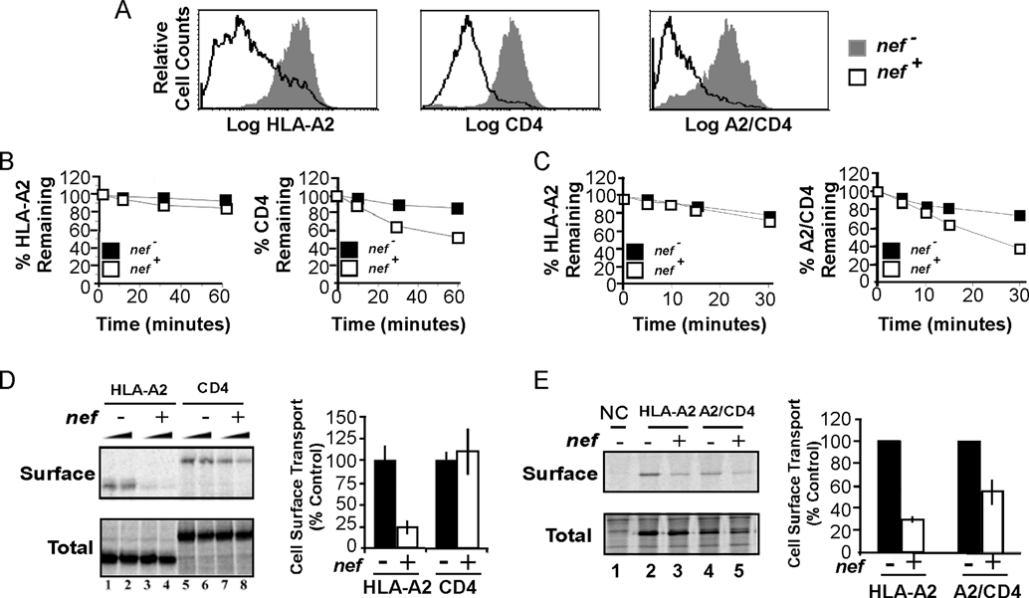
The cytoplasmic tail domain of MHC-I and CD4 determines the mechanism by which Nef affects trafficking. (A) Reduction of surface expression of HLA-A2, CD4 and A2/CD4 as measured by flow cytometry. CEM HA-HLA-A2 and CEM HA-A2/CD4 cells were transduced with a control adenovirus (*nef^−^*) or adeno-Nef (*nef^+^*) and stained for surface HLA-A2 and CD4. The histograms shaded gray represent cells treated with control adenovirus, and the solid black line indicates cells treated with adeno-Nef. (B-C) Measurements of surface stability. CEM HA-HLA-A2 and HA-A2/CD4 cells were treated with adenovirus as in part A and the internalization of endogenous CD4 (B) or A2/CD4 (C) was compared to the internalization of HLA-A2. The filled squares represent control (*nef−*) cells, and the open squares represent adeno-Nef (*nef^+^*) cells. Quantitation of part B was compiled from four independent experiments performed in duplicate. Part C is representative of two experiments performed in triplicate. (D–E) HLA-A2 is inefficiently transported to the cell surface in T cells expressing Nef. CEM HA-HLA-A2 and CEM HA-A2/CD4 cells were transduced as in part A. Metabolic labeling with continuous surface biotinylation was performed in the presence of a cell-impermeable biotinylation reagent [NHS-biotin, (Pierce)] to label cell surface proteins. The cells were lysed and immunoprecipitated first with an antibody against HLA-A2 or CD4 (part D) or anti-HA (part E), then 2/3 was re-precipitated with avidin beads to selectively precipitate the HLA-A2 on the cell surface. Normalized surface MHC-I was calculated as follows: ((surface MHC-I /total MHC-I×2)×100). Quantitation for parts D and E represents the mean±standard deviation for four and three independent experiments respectively. For part D, quantitation is derived from data for the lower of the two Nef levels shown.

It is known that Nef binds to the cytoplasmic tails of both CD4 and MHC–I, but that it affects them differently. To better understand the similarities and differences governing these two pathways, we examined the trafficking of CD4, HLA-A2 and a chimeric molecule in which the wild type HLA-A2 cytoplasmic tail was substituted with the CD4 cytoplasmic tail (HA-A2/CD4). A flow cytometric analysis of steady state surface expression revealed that Nef dramatically reduced steady state surface expression of all three molecules (Figure 1A). Consistent with prior studies, we found that CD4 was rapidly internalized from the cell surface in Nef expressing T cells, whereas wild type HLA-A2 was not (Figure 1B). Substitution of the CD4 tail for the HLA-A2 cytoplasmic tail was sufficient to confer this phenotype (Figure 1C). Conversely, prior studies have shown that Nef disrupts cell surface expression of MHC-I by blocking the transport of newly synthesized MHC-I from the TGN to the cell surface [22],[23]. As shown in Figure 1D, Nef inhibited HLA-A2 forward transport by approximately 75%, whereas CD4 was unaffected at Nef levels that had a clear effect on HLA-A2 transport. Slight effects on CD4 could be observed at higher Nef levels (Figure 1D, lane 8). The substitution of the HLA-A2 cytoplasmic tail with the CD4 tail reduced the ability of Nef to disrupt forward trafficking (Figure 1E). Thus, sequences in the cytoplasmic tails of CD4 and HLA-A2 determine how Nef disrupts their trafficking.

### CD4 and a subset of HLA-A2 proteins are found in late endosomes and lysosomes of Nef-expressing T cells

**Figure 2:**
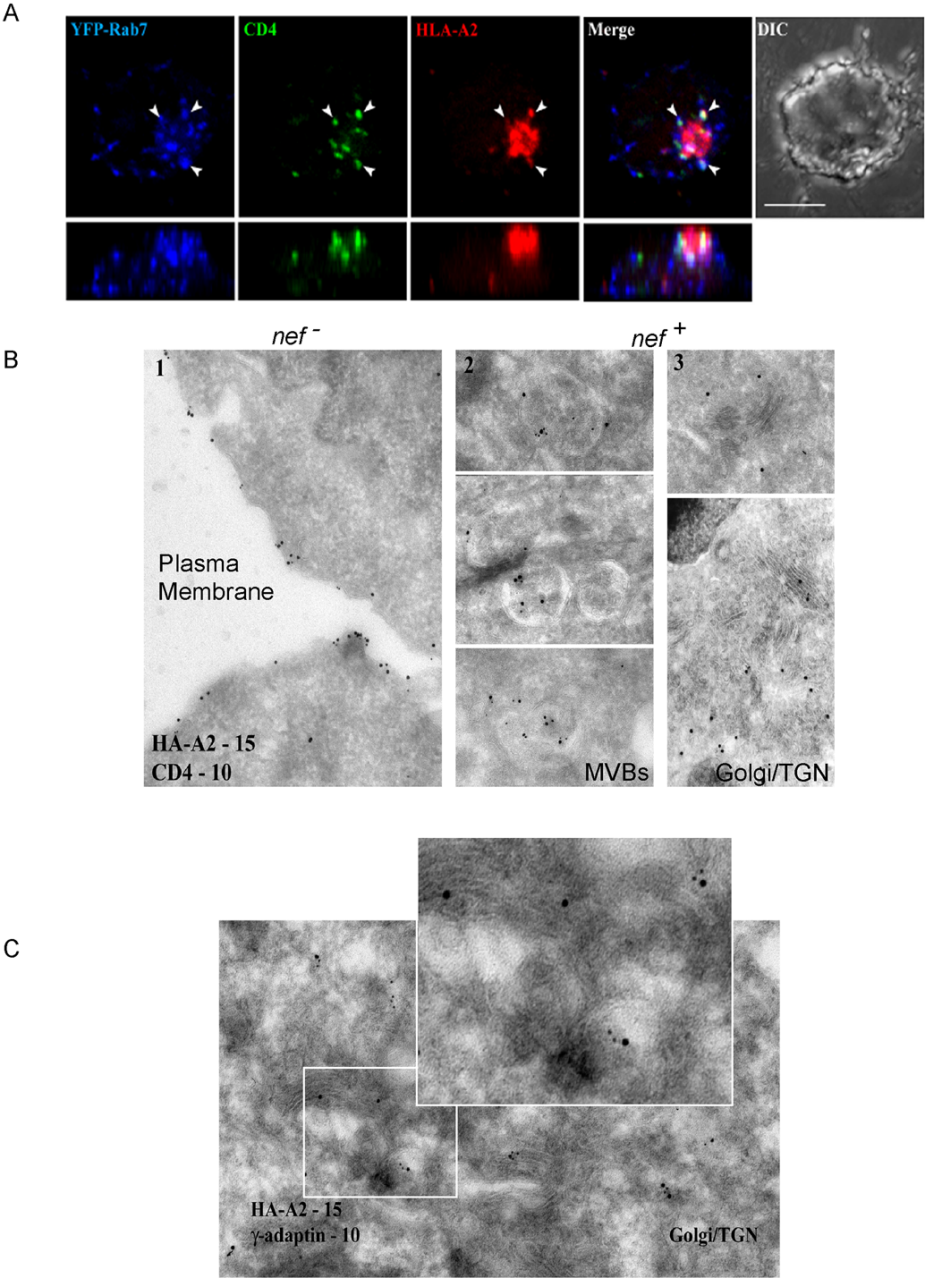
MHC-I and CD4 co-localize in a subset of vesicles in Nef-expressing T cells. (A) Three-way co-localization of HLA-A2, CD4 and Rab7. CEM HLA-A2 cells stably expressing YFP-Rab7 were transduced with adeno-Nef. HLA-A2 (red), CD4 (green), and YFP-Rab7 (blue) were simultaneously detected using three-color confocal microscopy. The top row shows the *x*–*y* projection of the cell, while the bottom row displays the *x*–*z* projection. Ten sequential optical sections were compiled to generate a projection of each cell about the *x*–*z* plane. Scale bar = 5 microns. (B) Immunogold labeling of HLA-A2 and CD4. Representative electron micrographs of CEM HA-HLA-A2 cells treated with control adeno (column 1) or adeno-Nef (columns 2 and 3). Thawed cryosections of cells were labeled with anti-HA (HLA-A2) and anti-CD4 antibodies followed by 15 and 10 nm protein A-gold respectively. (C) Immunogold labeling of HA-HLA-A2 and γ-adaptin in Nef-expressing CEM T cells. Thawed cryosections of cells were labeled with anti-HA (HLA-A2) and anti-γ-adaptin antibodies followed by 15 and 10 nm protein A-gold, respectively.

To better understand the similarities and differences between MHC-I and CD4 trafficking in Nef-expressing cells, we compared the steady-state distribution of these molecules in T cells using confocal microscopy (Figure 2A). We found that Nef expression caused the bulk of MHC-I to cluster in the perinuclear region where, in agreement with many other studies [14],[30],[49], it co-localized with markers of the TGN (data not shown). Interestingly, we also identified a subset of HLA-A2 that co-localized with CD4 in vesicular structures (Figure 2A; arrows show example vesicles). To further identify these structures, we simultaneously stained for HLA-A2, CD4, and organelle markers using 3-color confocal microscopy (summarized in Table S1). Our results indicated that CD4 was mainly found in discrete vesicular structures, which also contained HLA-A2 (91.9% of the CD4^+^ vesicles co-localized with HLA-A2, Table S1) and markers of late endosomes and lysosomes. Overall, the best marker for structures containing both HLA-A2 and CD4 was Rab7 (94%, of CD4+ vesicles co-localized with Rab 7, Table S1 and Figure 2A, arrowheads mark example vesicles). CD4 and HLA-A2 were also found to co-localize with markers of lysosomes, such as LAMP-1. However, the vesicles with the most intense LAMP-1 staining did not contain either HLA-A2 or CD4, possibly because of degradation. Consistent with this, the co-localization of HLA-A2 and CD4 was dramatically increased when the cells were treated with bafilomycin, which inhibits degradation in acidic compartments (Figure S1). Thus, the normal steady-state co-localization of HLA-A2 and CD4 in Nef expressing cells was limited because degradation prevented accumulation in this compartment.

### Colocalization of HLA-A2 and CD4 in MVBs

To further discern these structures, we also examined them using electron microscopy (EM). In agreement with the confocal data, our EM analysis revealed that compared with control cells in which both HLA-A2 and CD4 were found on the cell surface (Figure 2B, panel 1), in Nef-expressing T cells, the majority of CD4 was found in MVBs, co-localizing with HLA-A2 (Figure 2B, panel 2). In addition, we also noted substantial HLA-A2, but not CD4, accumulating in tubulovesicular structures adjacent to Golgi stacks (Figure 2B, panel 3). In separate experiments these structures were also found to contain AP-1 (Figure 2C). Based on these studies, it appears that the majority of HLA-A2 resides in tubulovesicular structures in the region of the TGN with AP-1, whereas at any given time, a small subset can be found in the endosomal compartment with CD4.

### Required cellular co-factors

To further elucidate the similarities and differences between these pathways, we examined the role of known Nef-interacting proteins implicated in intracellular trafficking. AP-1 is a heterotetrameric adaptor protein involved in protein sorting from the TGN and it has been previously demonstrated to interact with MHC-I molecules in Nef expressing HIV-infected primary T cells and to direct MHC-I into the endolysosomal pathway [25]. Nef is also known to interact with β-COP [46], a component of COP-1 vesicles also involved in endosomal trafficking [43]–[45]. Indeed, expression of wild type COP 1 components is needed for targeting CD4 into acidic vesicles in Nef-expressing cells [18].

**Figure 3:**
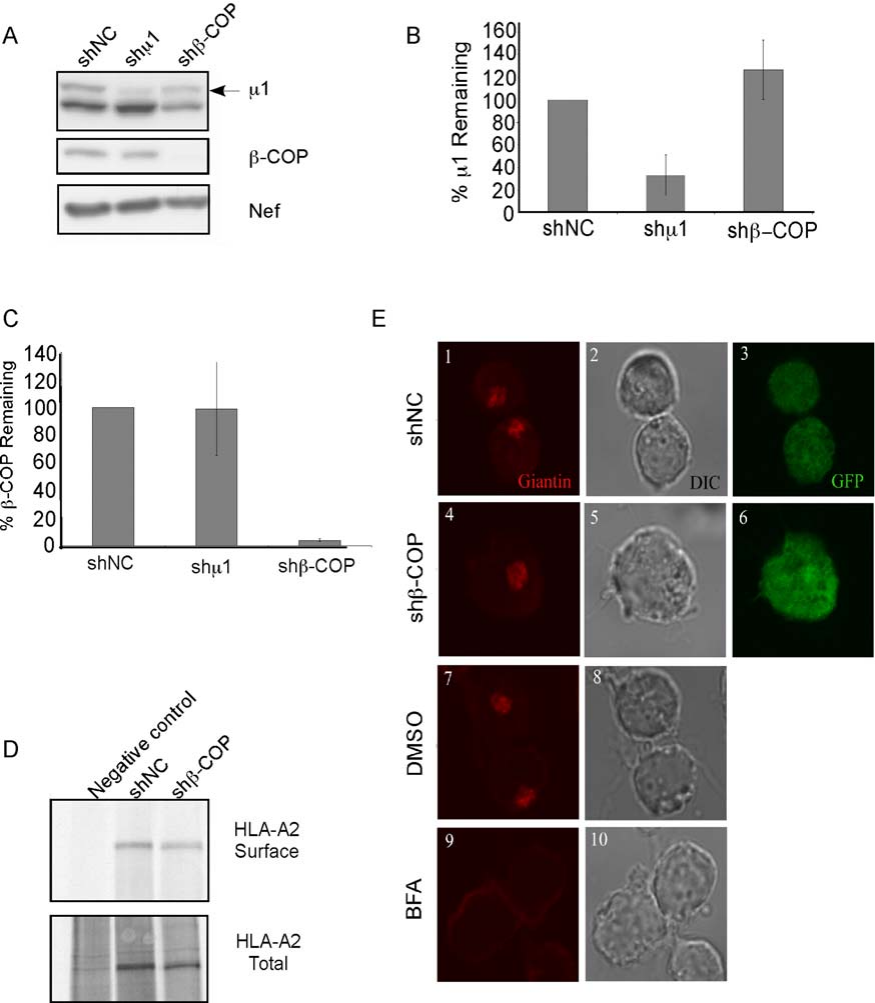
Knockdown of β-COP does not affect HLA-A2 transport to the cell surface or disrupt the Golgi apparatus. (A) Analysis of protein expression in β-COP and μ1 knockdown cells. CEM HA-HLA-A2 cells were transduced with a lentivirus expressing both GFP and a control shRNA (shNC) or an shRNA targeting either β-COP (shβ-COP) or μ1 (shμ1). At 72 hours later, they were transduced with adeno-Nef or control adenovirus. Three days later they were harvested, and western blot analysis was used to assess protein levels of β-COP, μ1 and Nef. (B,C) Quantification of μ1 and β-COP expression in shRNA treated cells. The amount of either μ1 (B) or β-COP (C) was quantified using Adobe Photoshop software. The average percent remaining±standard deviation for four experiments (B) and three experiments (C) is shown. To adjust for protein loading in part B, the nonspecific background band directly below μ1 (shown in part A) was used to normalize protein loading. (D) Knockdown of β-COP does not affect HLA-A2 transport to the cell surface. CEM HA-HLA-A2 cells were transduced with lentivirus expressing either shNC or shβ-COP as in part A. Cell surface transport was assessed using a metabolic labeling assay with biotinylation as described in Figure 1D. (E) Knockdown of β-COP does not disrupt the Golgi apparatus. CEM HA-HLA-A2 cells were transduced with lentivirus expressing the indicated shRNA and GFP as in part A and treated with brefeldin A (BFA) at 50 µM or DMSO for 30 minutes. The integrity of the Golgi apparatus was assessed by immunofluorescence staining for giantin and analyzed by confocal microscopy. Images were taken using a Zeiss confocal microscope and analyzed with LSM Image Browser and Adobe Photoshop software. Single Z-sections are shown. The results shown for parts D and E are representative of three independent experiments.

To compare and contrast the requirement for these factors in Nef-dependent CD4 and HLA-A2 trafficking, we knocked down their expression using lentiviral vectors expressing short hairpin RNAs (shRNAs) [50]. All of these studies were performed in T cells and new cell lines were generated for each experiment to eliminate the possibility that long term growth in culture would select for cells that had compensated for the defect. Using this system, we obtained good knock down of the μ1 subunit of AP-1 and β-COP (Figure 3A–C). (A small apparent effect of shβ-COP on μ1 levels observable in Figure 3A was not significant when adjusted for protein loading in the experiment shown here or in replicate experiments [Figure 3B]. We also did not observe any effect of another siRNA directed against a different target site in β-COP on μ1 expression [Figure S2].)

### The effect of knocking down β-COP expression on the structural integrity of the Golgi

Because β-COP is known to be important for intra-Golgi and ER-to-Golgi trafficking, we asked whether the Golgi structure or MHC-I trafficking were drastically affected by reduced β-COP expression. We found that there was only a small reduction in the normal transport of MHC-I to the cell surface (35% reduction, Figure 3D). In addition, cells lacking β–COP generally maintained overall Golgi structure as assessed by the intracellular localization of giantin, a transmembrane protein normally residing in the *cis* and *medial* Golgi [51] (Figure 3E). In contrast, brefeldin A, an inhibitor of an ARF1 GEF necessary for β-COP activity obliterated the normal Golgi staining (Figure 3E, panel 9). The relatively mild phenotype of this knock-down compared to the drastic effects of brefeldin A, suggests that brefeldin A has effects other than just disrupting COP 1 coats by blocking ARF1 activity.

**Figure 4:**
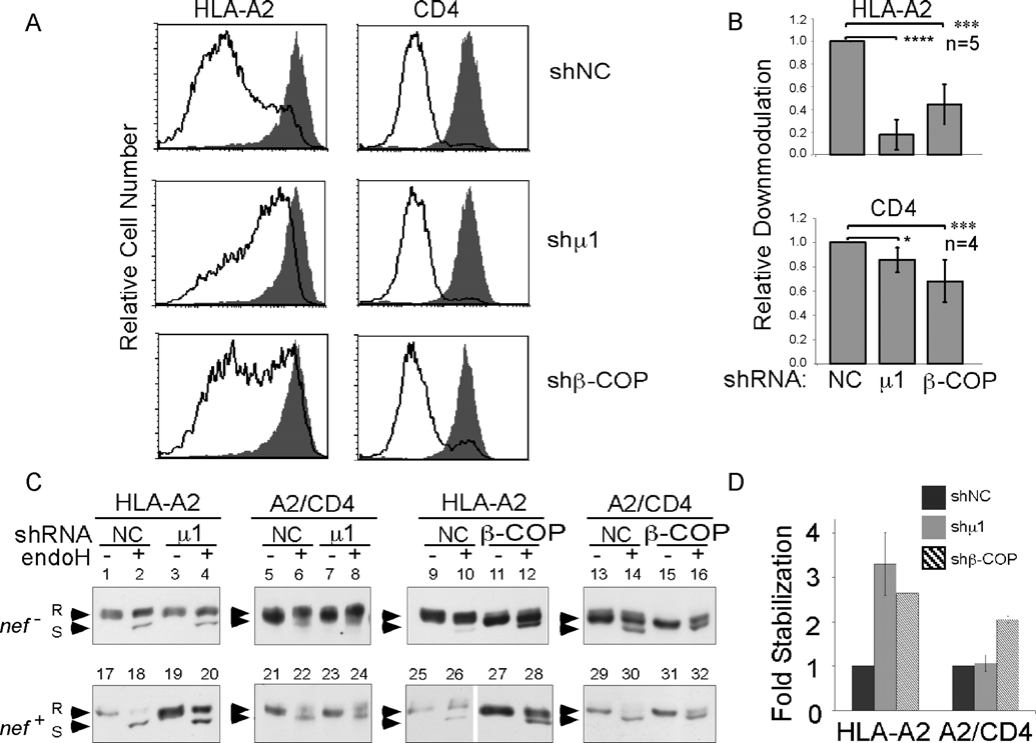
Nef requires β-COP to reduce HLA-A2 cell surface expression and accelerate HLA-A2 degradation. (A) β-COP and μ1 are required for Nef to reduce cell surface expression of HLA-A2. CEM HA-HLA-A2 cells were transduced with a lentivirus expressing GFP and a control (shNC), β-COP (shβ-COP) or μ1 (shμ1) shRNAs and with control adenovirus (*nef^−^*) or adeno-Nef (*nef^+^*). Cell surface expression of HLA-A2 or CD4 in the GFP-positive cells was assessed by flow cytometry. The gray shaded histogram represents control adenovirus (*nef^−^*) treated cells and the solid black line represents adeno-Nef (*nef^+^*) treated cells. (B) Quantitation of HLA-A2 and CD4 downmodulation in Nef expressing cells transduced with shRNA. The median fold downmodulation (median fluorescence of control/median fluorescence of Nef-expressing cells)±standard deviation derived from five (HLA-A2) and four (CD4) independent experiments. A *p* value was calculated using a two tailed t-test and significant differences were indicated with asterisks (*p<0.02, ***p<10^−3^, ****p<10^−4^). (C) Knockdown of β-COP stabilizes intracellular levels of HLA-A2 and A2/CD4 in Nef expressing cells. CEM HA-HLA-A2 and CEM HA-A2/CD4 were treated as in part A. Lysates from these cells were generated and treated with endoglycosidase H (endo H). Protein levels of HLA-A2 and A2/CD4 were assessed by western blot using an anti-HA antibody. Endo H–resistant bands are marked with an R and endo H–sensitive bands are marked with an S. The results shown are representative of three independent experiments for HLA-A2 and two independent experiments for A2/CD4. (D) Quantification of endo H–resistant protein. Adobe Photoshop software was used to quantify each band for the Nef-expressing samples. The percentage of endo H–resistant protein in each condition was calculated as follows: [resistant band/(resistant band+sensitive band)]×100. The fold stabilization was then calculated as: (% endo H–resistant in experimental sample)/[% endo H–resistant in control (shNC)]. The data shown is the mean of two experiments±standard deviation.

Having established that knocking down β-COP allowed relatively normal forward trafficking of HLA-A2, we proceeded to assess the effect of knocking down β-COP or AP-1 in Nef-expressing cells. Consistent with previous publications [25], we found that knocking down the ubiquitously expressed form of AP-1 (AP-1A [52]) largely reversed the effect of Nef on HLA-A2 (p<10^−4^), but had a smaller and less significant effect (*p*<0.02) on CD4 surface expression (Figure 4A and 4B). Surprisingly, we also observed that knocking down β-COP expression inhibited MHC-I downmodulation by Nef and had a small but statistically significant effect on CD4 downmodulation (p<10^−3^; Figure 4A and 4B). The small effect of β-COP on CD4 surface expression indicated that β-COP was not necessary for CD4 internalization and downmodulation from the cell surface. However, further studies were needed to determine whether β-COP was required to degrade the CD4 after it was internalized.

### A role for β-COP in promoting degradation of Nef cellular targets

Prior studies had determined that expression of β-COP was necessary for acidification of CD4-containing vesicles and thus it was hypothesized that β-COP was needed to target vesicles containing internalized CD4 for lysosomal degradation. Therefore, we asked whether the role of β-COP in MHC-I trafficking was also to promote MHC-I degradation. To examine this, we utilized an assay we had developed, which measures the loss of mature, endo H–resistant HA-tagged HLA-A2 in Nef expressing cells by western blot analysis. This assay system is based on previous data demonstrating Nef-dependent degradation of the mature form of MHC-I in a manner that is reversible by inhibitors of lysosomal degradation [25]. As shown in Figure 4C, under normal, steady state conditions, most of the HLA-A2 is resistant to endo H digestion, indicating that it has matured through the Golgi apparatus (Figure 4C, lane 2). However, when Nef was expressed, we observed a dramatic reduction in total MHC-I and a decrease in the ratio of endo H resistant to sensitive protein (Figure 4C compare lanes 2 and 18, see also Figure S3). Consistent with a role for AP-1, we observed that AP-1A shRNA largely reversed this effect of Nef (Figure 4C, compare lanes 18 and 20. See also Figure 4D for quantification). To detect degradation of molecules containing a CD4 tail, we used HA-A2/CD4 (Figure 1) and found that Nef expression accelerated the degradation of endo H resistant forms of this molecule (Figure 4C, compare lanes 6 and 22). However, we found that there was no effect of reduced AP-1A expression on Nef-dependent degradation of molecules containing the CD4 tail (Figure 4C, compare lanes 22 and 24. See also Figure 4D for quantification).

When β-COP expression was reduced, we observed a small increase in the amount of immature, endo H–sensitive protein (Figure 4C, compare lanes 10 and 12), consistent with the 35% reduction in export of MHC-I to the cell surface shown in Figure 3D. However, we also noted that reduction in β-COP expression reduced the Nef-dependent degradation of the mature, endo H resistant form of these molecules (Figure 4C, compare lanes 26 and 28. See also Figure 4D for quantification) implicating β-COP in this pathway. We were also able to confirm the model that β-COP is involved in Nef-dependent CD4 degradation as treating cells with β-COP shRNA reduced the degradation of the A2/CD4 chimeric molecule (Figure 4C, compare lanes 30 and 32. See also Figure 4D for quantification).

### β-COP is required for targeting internalized CD4 for degradation in Nef-expressing T cells

**Figure 5:**
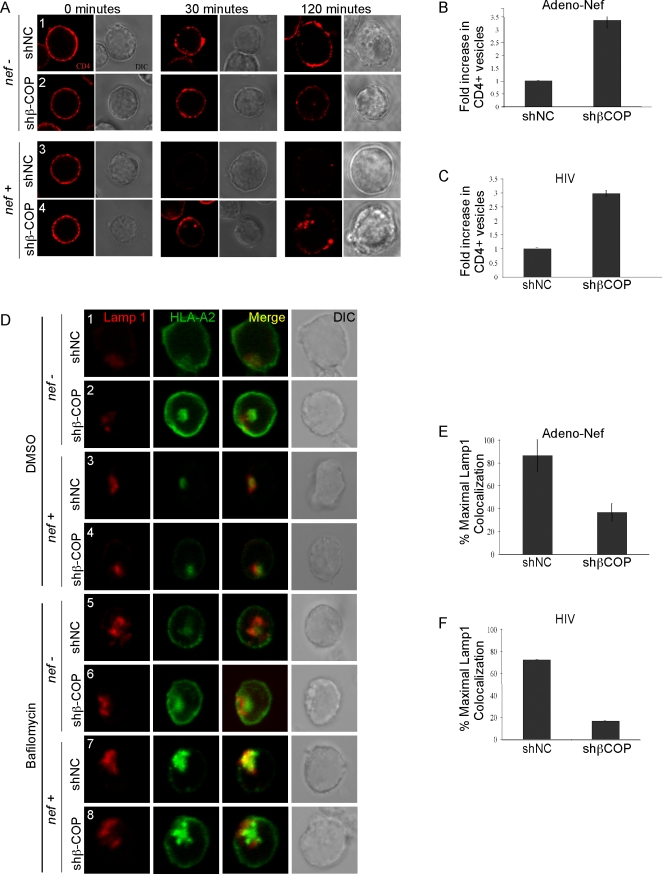
Nef requires β-COP to target HLA-A2 and CD4 for degradation. (A) Knockdown of β-COP stabilizes CD4^+^ vesicles in Nef expressing cells. CEM HA-HLA-A2 cells transduced with a lentivirus expressing GFP and either control shRNA (shNC) or shRNA targeting β-COP (shβ-COP) were transduced with control adenovirus (*nef^−^*) or adeno-Nef (*nef^+^*). The cells were incubated with CD4 antibody on ice and then shifted to 37°C for internalization for the indicated times. Images were taken with a Zeiss confocal microscope and processed using LSM Image Browser and Adobe Photoshop software. Single Z-sections are shown. (B) Quantitation of CD4^+^ vesicles is shown for 15 GFP^+^, *nef* ^+^ cells treated with shNC and 17 GFP^+^, *nef* ^+^ cells treated with shβ-COP. The mean±standard deviation is shown. (C) Quantitation is shown for 5 GFP^+^, *nef* ^+^ cells treated with shNC and 5 GFP^+^, *nef* ^+^ cells treated with shβ–COP. The mean±standard deviation is shown. (D) CEM HA-HLA-A2 cells were transduced with a lentivirus expressing either GFP and control (shNC) or β-COP (shβ–COP) shRNA, infected with HIV, treated with bafilomycin or DMSO and stained for HLA-A2 and LAMP-1 as previously described [25]. Images were taken with a Zeiss confocal microscope and processed as in part A. Single Z-sections are shown. (E) Relative co-localization of HLA-A2 with LAMP-1 in 10 GFP^+^, adeno-Nef-expressing T cells treated with shNC and 15 GFP^+^, adeno-Nef-expressing T cells treated with shβ-COP. (F) Relative co-localization of HLA-A2 with LAMP-1 in 6 GFP^+^, HIV-*nef* ^+^ infected T cells treated with shNC and, 6 GFP^+^, HIV-*nef* ^+^–infected T cells treated with shβ-COP. Quantitation of microscopy data was performed independently by two blinded investigators who scored maximal observable co-localization among all cells at an arbitrary value of 5. Each cell was then scored relative to that. The mean±standard deviation is shown.

We next directly examined the effect of reducing β-COP expression on Nef-dependent trafficking by confocal microscopy. For these experiments, cells were infected with HIV or were transduced with Nef-expressing adenoviral vectors and then the fate of internalized CD4 was assessed by confocal microscopy. Using this assay system, we observed fairly rapid internalization of CD4 in Nef-expressing cells, followed by loss of CD4 staining by 30 minutes (Figure 5A, compare control cells in row 1 to Nef-expressing cells in row 3). However, in T cells expressing β-COP shRNA, there was a three-to-four fold increase in the number of CD4-containing vesicles, consistent with a role for β-COP in promoting maturation of these vesicles into degradative compartments (Figure 5A, compare control treated Nef-expressing cells in row 3 to shβ-COP–expressing cells in row 4). Reduction of β-COP expression yielded similar results whether Nef was introduced using HIV infection or via adenoviral vectors (Figure 5B and 5C).

### β-COP is required for targeting MHC-I to LAMP-1^+^ compartments in Nef-expressing T cells

Confocal analysis of MHC-I intracellular localization revealed that expression of β-COP shRNA in control cells increased the intracellular accumulation of MHC-I, consistent with the slowing of export we observed in cells deficient in β-COP (Figure 5D, compare rows 1 and 2). Infection with Nef-expressing HIV resulted in the loss of cell surface MHC-I and an increase in intracellular MHC-I, some of which co-localized with LAMP-1 (Figure 5D, compare rows 1 and 3). Under these conditions, reduction of β-COP expression reduced the degree of colocalization with LAMP-1 (Figure 5D, compare rows 3 and 4).

To enhance our ability to observe trafficking of MHC-I into LAMP-1^+^ compartments, we treated the cells with bafilomycin, which inhibits the vacuolar ATPase and thus acidification and degradation within lysosomal compartments. As previously reported [25], bafilomycin treatment enhanced our ability to detect MHC-I in LAMP-1^+^ compartments in Nef-expressing T cells (Figure 5D, compare rows 3 and 7). The expression of β-COP shRNA decreased LAMP-1 colocalization with MHC-I, consistent with a role for β-COP in targeting MHC-I for degradation in lysosomal compartments in Nef expressing T cells (Figure 5D, compare rows 7 and 8). Similar results were observed whether Nef was introduced using HIV or adenoviral vectors (Figure 5E and 5F).

We also examined co-localization of HLA-A2 and CD4 in cells that expressed β-COP shRNA. We observed that reduction of β-COP expression resulted in increased staining of both proteins, and did not disrupt their co-localization (Figure S4). Thus, β-COP was not necessary for targeting these proteins into a common endosomal pathway, but rather was needed for their subsequent targeting into a degradative pathway.

### The cytoplasmic tail of MHC-I is necessary for AP-1 binding in Nef-expressing T cells

**Figure 6:**
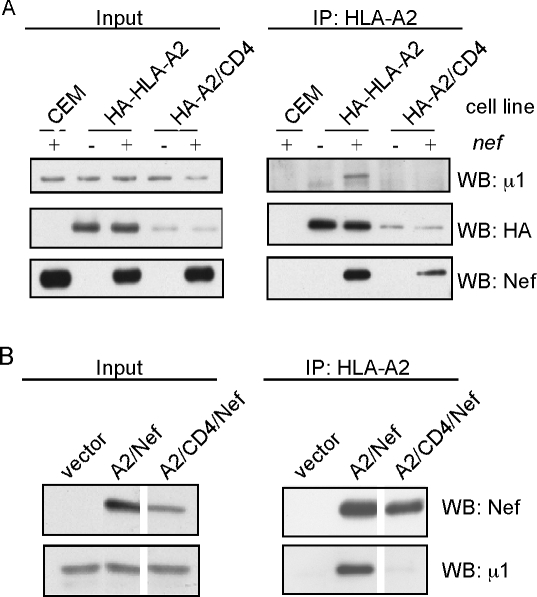
Selective binding of AP-1 is dependent on the cytoplasmic tail. (A) The HLA-A2 cytoplasmic tail is necessary for co-precipitation of AP-1. Parental HLA-A2-negative CEM T cells (CEM) or CEM T cell lines expressing HA-HLA-A2 or HA-A2/CD4 were transduced with adeno-Nef or a control adenovirus. Lysates were immunoprecipitated with an antibody directed against HLA-A2 (BB7.2) and the presence of Nef or AP-1 was detected by western blot analysis. Results are representative of three independent experiments. (B) The cytoplasmic tail is necessary for the HLA-A2/Nef fusion protein to co-precipitate AP-1 in Nef expressing T cells. CEM T cells were transduced with a murine retroviral vector expressing no protein (vector), A2/Nef or A2/CD4/Nef fusion proteins. These cells were immunoprecipitated with an anti-HLA-A2 antibody (BB7.2) and western blot analysis was performed to detect co-precipitation of AP-1. Spaces between lanes indicate where intervening lanes were cropped out to remove irrelevant data. Results are representative of two independent experiments.

To further explore the molecular mechanism for the similarities and differences in MHC-I and CD4 trafficking in Nef-expressing T cells, we asked whether these molecules differed as to how well they bound Nef or cellular factors. As expected, we found that HIV Nef bound to both the HLA-A2 and the CD4 tail (Figure 6A, right panel). However, AP-1 only co-precipitated with molecules containing the HLA-A2 cytoplasmic tail (Figure 6A, right panel). The chimeric molecule with the CD4 cytoplasmic tail did not bind AP-1 in Nef-expressing T cells (Figure 6A, right panel). In these experiments, we noted that the expression level of A2/CD4 was lower than for wild type HLA-A2, which could explain this difference. Therefore, we confirmed these data using a fusion protein containing either HLA-A2 or A2/CD4 directly fused to full length HIV-Nef protein. In previously published experiments it was shown that the HLA-A2/Nef fusion protein co-precipitated AP-1 in a manner that depended on sequences both in Nef and in the HLA-A2 cytoplasmic tail [25]. Here we show again that the HLA-A2 cytoplasmic tail was necessary for this interaction and, moreover, that the CD4 tail could not substitute for it (Figure 6B, right panel).

### Evidence that formation of a Nef–β-COP complex is an essential step necessary for MHC-I degradation

**Figure 7:**
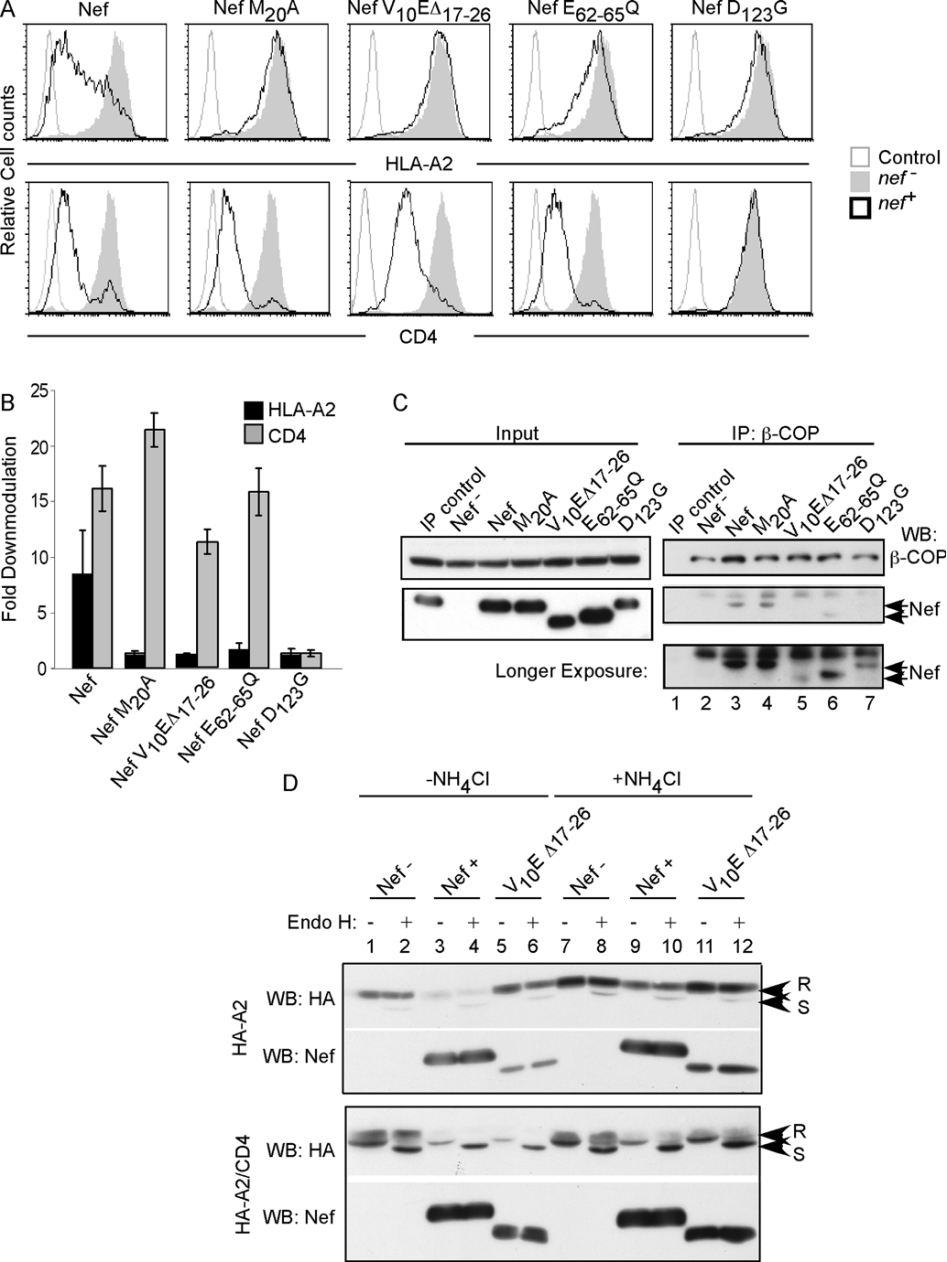
Co-precipitation of Nef and β-COP depends on domains of Nef that are also needed for MHC-I downmodulation. (A) Flow cytometric analysis of Nef mutants defective at MHC-I downmodulation. CEM T cells treated with control adenovirus (*nef^−^*), adeno-Nef (*nef^+^*) or the indicated mutant were stained either with an anti-HLA-A2 antibody (BB7.2) or an antibody directed at CD4. Cells were analyzed by flow cytometry as described in Materials and Methods. (B) Quantitation of MHC-I and CD4 downmodulation by Nef and Nef mutants. Fold downmodulation was determined by dividing the mean fluorescence intensity (MFI) of control virus treated cells by the MFI of Nef-expressing cells. The average value from three (wild-type) or two (mutant Nef) experiments was plotted±the standard deviation. (C) Nef D_123_G and V_10_EΔ17–26 mutants are defective at β-COP binding. CEM T cells were treated with control adenovirus (*nef^−^*), adeno-Nef (*nef^+^*), or the indicated mutant and immunoprecipitated with a control antibody (BB7.2) or an antibody directed against β-COP (M3A5). The presence of Nef was detected by western blot analysis. Arrows indicate the positions of wild type Nef and Nef V_10_EΔ17–26. Results are representative of at least two independent experiments. (D) V_10_EΔ17–26 Nef is defective at MHC-I, but not CD4, degradation. CEM cells expressing HA-HLA-A2 and HA-A2/CD4 were transduced with adeno-viral vectors encoding wild-type Nef (Nef^+^), V_10_EΔ17–26 Nef, or a control adenoviral vector (Nef^−^). Two days later, the media on half of the cells was replaced with media containing 20 mM ammonium chloride to inhibit lysosomal degradation. The next day, the cells were harvested, lysed, and normalized. Each sample was split equally and one set was treated with endo H. Protein levels of HA-HLA-A2 and HA-A2/CD4 were assessed by western blot analysis using an anti-HA antibody. Endo H–resistant bands are marked with an R and endo H–sensitive bands are marked with an S.

The Nef-β-COP interaction is well-described in the literature [46] and there is evidence that β-COP interacts with a diacidic motif (E_154/155_) within the Nef C-terminal loop [18]. However, this region of Nef has never been implicated in MHC-I trafficking. To provide further evidence that β-COP is needed to promote MHC-I degradation, we sought to identify a region of Nef that is needed both for MHC-I degradation as well as β-COP binding. We therefore examined a panel of mutations (M_20_A, V_10_EΔ17–26 and E_62–65_Q) that are specifically defective at disrupting MHC-I trafficking [14],[15],[26],[53]. We also examined a Nef mutant, D_123_G, that is defective at both CD4 and MHC-I downmodulation [21]. The relative activity of these Nef mutants in MHC-I and CD4 downmodulation is shown in Figure 7A and quantified in Figure 7B.

We then examined the relative ability of each of these mutant molecules to co-precipitate with β–COP. As shown in Figure 7C, we found that the V_10_E Δ17–26-Nef, which is defective at MHC-I downmodulation, was also defective at binding to β-COP (compare lanes 3 and 5). Interestingly, this deletion mutant is also defective at interacting with AP-1 [25]. However, the β-COP binding site was separable from the AP-1 interaction site because M_20_, which is located within the deleted region, is needed for AP-1 interaction [25],[27]), but was not necessary for β-COP binding to Nef (Figure 7C, compare lanes 3 and 4). Mutation of the Nef dimerization motif [D_123_G, [21]], which disrupts a number of Nef functions, including MHC-I and CD4 downmodulation, also reduced binding to β-COP (Figure 7C, compare lanes 3 and 7). Finally, mutation of the Nef acidic domain (E_62–65_Q), which disrupts binding to MHC-I [26], AP-1 [27],[28] and PACS-1 [54], did not affect binding to β-COP (Figure 7, compare lanes 3 and 6).

As expected, we found that V_10_EΔ_17–26_ Nef, which was defective at β-COP binding, was also defective at inducing the degradation of the endo H resistant form of HLA-A2 (Figure 7D, upper panel, compare lanes 3 and 4 with lanes 5 and 6). In contrast, V_10_EΔ17–26 Nef was not defective at A2/CD4 degradation based on western blot analysis (Figure 7D, lower panel, compare lanes 3 and 4 with lanes 5 and 6). These data suggested that there may be another interaction domain that recruits β-COP to the Nef-CD4 complex to promote CD4 degradation. This would be consistent with the faint band observable in the V_10_EΔ_17–26_-Nef mutant immunoprecipitation (Figure 7C, lane 5, longer exposure) and prior publications demonstrating that mutation of E_154/155_ also affected β-COP binding [47]. Thus, there may be two independent binding sites for β-COP within Nef, each of which governs the degradation of a different cellular factor.

**Figure 8:**
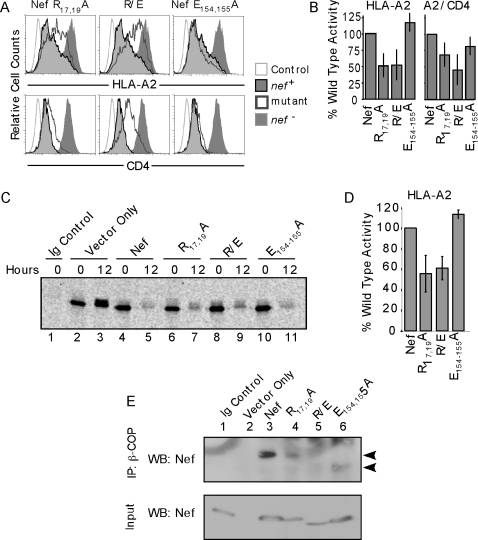
Two Nef domains recruit β-COP, but only one is used for the degradation of HLA-A2. (A) Flow cytometric analysis of HLA-A2 and CD4 expression in cells expressing Nef mutants. CEM T cells stably expressing HLA-A2 were spin-transduced with murine retroviral supernatants that express the indicated Nef construct and a GFP cassette from an internal ribosomal entry site. The cells were gated for GFP expression and results of HLA-A2 (top panel) or endogenous CD4 (bottom panel) staining are shown. R/E stands for R_17,19_A/E_154,155_A double mutant. Open light gray curve, parental cell line; shaded dark gray curve, empty vector; black shaded curve, wild type Nef; and open dark gray curve, Nef mutant. (B) Quantification of down-modulation. The mean±SD for greater than or equal to six experiments (actual number varies depending on the mutant) is shown. (C,D) R_17/19_ is needed for optimal HLA-A2 degradation, whereas the E_154/155_ is dispensable. CEM T cells expressing HLA-A2 and Nef were generated as described in part A. The cells were pulse labeled with ^35^S-labeled amino acids, chased for 0 or 12 hours in complete medium and lysed. HLA-A2 was immunoprecipitated with the anti-HLA-A2 antibody BB7.2, separated by SDS-PAGE and quantified using a phosphorimager. “Ig Control” indicates results from HLA-A2-negative parental CEM cells immunoprecipitated with BB7.2 antibody. (D) Quantification of degradation. Nef activity was calculated as follows: (the fraction of HLA-A2 remaining in control cells /the fraction of HLA-A2 remaining in Nef expressing cells). The value obtained for each mutant was divided by that for wild type Nef and multiplied by 100 to calculate % wild type activity. The mean±SD for two experiments is shown. (E) R_17/19_ and E_154/155_ are required for the β-COP/Nef interaction. CEM T cells expressing HA-A2 were transduced with a retroviral vector expressing either wild-type Nef or the indicated Nef mutant. The cells were immunoprecipitated with an anti–β-COP antibody and the presence of Nef was assessed by western blot as described in Materials and Methods. The Ig control is HLA-A2–negative parental CEM cells expressing wild-type Nef immunoprecipitated with a control antibody (BB7.2) and the vector only control is CEM cells expressing HLA-A2 transduced with the empty retroviral vector.

To further define the β-COP binding site, and to determine whether there were indeed two β-COP binding sites, we constructed additional Nef mutants. We focused on the arginine residues (R_17_ER_19_MR_21_R_22_) within the Nef deletion Δ17–26) because previous studies had indicated that arginine rich regions could form β-COP-binding sites [55]. Flow cytometric analysis of MHC-I levels on cells expressing these mutants revealed that the R_17/19_ pair was necessary for maximal MHC-I downmodulation (Figure 8A and 8B). In contrast, mutation of R_21/22_ did not significantly affect MHC-I downmodulation (unpublished data). An assessment of Nef-induced degradation by pulse chase analysis of HA-HLA-A2, revealed that mutating this motif also inhibited Nef-dependent degradation (Figure 8C, compare lanes 5 and 7, quantified in Figure 8D). Additionally, mutation of R_17/19_ reduced, but did not eliminate binding of β-COP to Nef in a manner similar to the effect of the Δ17–26 Nef mutation (Figure 8E, compare lanes 3 and 4).

We next examined the diacidic motif (E_154/155_) previously implicated in β-COP binding. As shown in Figure 8A and 8B, mutation of this motif did not disrupt MHC-I downmodulation, in fact downmodulation was somewhat enhanced. Additionally, we found that mutation of this motif did not reduce MHC-I degradation (Figure 8C, compare lanes 5 and 11, see also quantification in 8D). However, in agreement with prior results, we observed a partial defect in β-COP binding with this mutant (Figure 8E, compare lanes 3 and 6, [18],[47]. However, this defect was less reproducible (observed in two out of four experiments) than that observed with disruption of R_17/19_ (consistently observed in five out of five experiments), suggesting that binding to R_17/19_ can mask the defect observed with mutation of E_154/155_ under certain conditions. To provide additional data supporting the possibility that both sites contributed to β-COP binding, we constructed a double mutant, R_17/19_ A and E_154/155_A (R/E). As shown in Figure 8E, lane 5, binding of R/E to β-COP was further reduced relative to binding of Nef proteins containing single mutations in each motif, strongly implicating both motifs in β-COP binding. The phenotype of the double mutant was highly reproducible in 5 out of 5 experiments.

**Figure 9:**
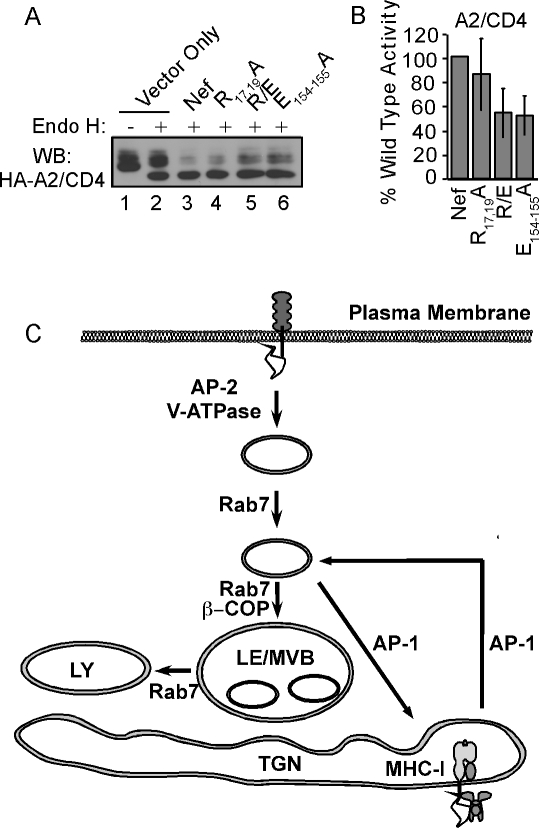
Nef uses the E_154/155_ to promote maximal CD4 degradation. (A) Cells expressing HA-HLA-A2/CD4 were treated as in Figure 8A, lysed and treated with endo H as indicated. The samples were separated by SDS-PAGE and western blotted for the HA tag on HA-A2/CD4. (B) Quantification of degradation. Western blots were quantified using Adobe Photoshop software. Nef activity was calculated as follows (fraction of total protein that was endo H–resistant for wild type Nef/fraction endo H–resistant for each mutant)×100. The mean±SD for four experiments is shown. (C) Model for the mechanism by which Nef affects CD4 and MHC-I trafficking. HIV Nef binds the CD4 cytoplasmic tail at the cell surface, and recruits AP-2 and/or the vacuolar-ATPase to facilitate internalization. CD4 is internalized and is transported to an endosomal compartment associated with Rab7 and β-COP. In contrast, Nef binds the MHC-I cytoplasmic tail early in the secretory pathway, AP-1 is recruited and facilitates transport to an intermediate endosomal compartment marked with Rab7. If AP-1 falls off the Nef-MHC-I complex after arrival in the endosome, Nef binds β-COP and targets MHC-I (and CD4) to lysosomes for degradation. If AP-1 remains bound, it promotes recycling of the Nef-MHC-I complex to the TGN. LY = lysosome, LE/MVB = late endosome/multi-vesicular body.

Interestingly, the R/E double mutant was not more defective than R_17/19_A at downmodulating MHC-I (Figures 8A and 8B) or at promoting MHC-I degradation (Figure 8C, compare lanes 7 and 9, quantified in 8D), indicating that Nef did not utilize the E_154/155_ binding site to recruit β-COP for MHC-I degradation. Conversely, we confirmed prior reports that the E_154/155_A mutant was defective at CD4 degradation (Figure 9A, compare lanes 3 and 6) and determined moreover that there was no significant effect of mutating R_17/19_ on CD4 degradation, either alone or in combination with E_154/155_A (Figure 9A, compare lanes 3 and 4). It is also worth noting that, in contrast to what was observed with HLA-A2, we did not observe a clear correlation between the relative CD4 surface expression and the relative level of total cellular CD4 (compare Figure 8B and 9B), indicating that there was a complex relationship between total cellular CD4 and the fraction expressed on the cell surface.

Because the R_17/19_ motif is directly adjacent to M_20_, which is necessary for AP-1 recruitment [25],[27], we also examined whether these mutations, which affect β-COP binding, also disrupted AP-1 co-precipitation. To accomplish this, we used our standard AP-1 recruitment assay in which proteins co-precipitating with MHC-I HLA-A2 were detected by western blot analysis. As shown in Figure S5, mutation of R_17,19_ (and E_154/155_) decreased AP-1 binding only slightly. Thus, the defects in MHC-I downmodulation and degradation noted with mutation of R_17,19_ resulted primarily from defects in β-COP binding.

## Discussion

Expression of HIV Nef in infected cells protects them from lysis by CTLs and this activity of Nef is due to downmodulation of MHC-I surface expression. The Nef protein also prevents superinfection and promotes viral spread by removing the viral receptor, CD4 from the cell surface (for review see [56]). We provide evidence that sequences in the cytoplasmic tail of these molecules are important for determining whether Nef disrupts their trafficking from the cell surface or at the TGN. These data, that swapping cytoplasmic domains switches the initial pathways taken by HLA-A2 and CD4 in the presence of Nef, may seem somewhat obvious. Nef is always the same and thus one might conclude that this information has to be contained in the modulated protein. However, it was also possible that the ectodomain affected Nef responsiveness by binding to other transmembrane proteins or by altering intracellular trafficking. This was certainly a possibility for MHC-I for which it is clear that the efficiency of peptide loading can affect trafficking and we have found that trafficking rates affect responsiveness to Nef and AP-1 binding [23].

Prior studies have demonstrated that Nef initially binds to hypo-phosphorylated forms of the MHC-I cytoplasmic tail early in the secretory compartment [23], but binding does not affect normal transit through the Golgi apparatus and into the TGN [25]. The Nef-MHC-I complex then recruits the AP-1 heterotetrameric clathrin adaptor protein using a binding site that is created when Nef binds the MHC-I cytoplasmic tail. This binding site requires a methionine from the N-terminal α helix of Nef and a tyrosine residue in the MHC-I cytoplasmic tail [25]. Additionally, there is evidence that this complex is stabilized by the acidic and polyproline domains of Nef [27],[28]. Formation of this complex results in the re-direction of MHC-I trafficking in such a way that it is targeted to lysosomes for degradation [25]. However, cellular proteins that normally bind AP-1 are not degraded, but rather recycled to the TGN (Figure 9C). Here we present new evidence that Nef utilizes β-COP to promote trafficking to degradative compartments (Figure 9C). Knocking down expression of β-COP inhibited the degradation of MHC-I and it did so by blocking the transport of MHC-I from intracellular vesicles to LAMP-1^+^ compartments. We also provide results here that confirm β-COP is necessary for degradation of CD4 in lysosomal compartments. Thus, we propose that AP-1 and AP-2 deliver MHC-I and CD4 respectively to endosomal compartments where β-COP displaces AP-1 and AP-2 to target MHC-I and CD4 for lysosomal degradation (Figure 9C).

As described above, we found that knocking down β-COP with shRNA resulted in stabilization of internalized CD4, however the effect on CD4 surface expression was small, but still significant. In contrast, there was a greater effect of β-COP knockdown on HLA-A2 surface expression. This might suggest that the role of β-COP in the modulation of these targets was different, rather than the same. However, this apparent paradox can be explained by our model shown in Figure 9C. As indicated, differences in response to β-COP knockdown can be explained by differences in the intracellular pathways of these proteins before they interact with β-COP. MHC-I is engaged in an AP-1-dependent endosome-to-TGN loop, and MHC-I could “leak” out to the cell surface from the TGN in the absence of β-COP, whereas CD4 may be unable to return to the cell surface from its endosomal compartment. Consistent with this, we also noted a lack of correlation between degradation and surface expression of CD4 (but not MHC-I) when Nef mutants that were defective in β-COP binding were examined. These data indicate that there is a complex relationship between total cellular CD4 and the fraction that is present on the cell surface and thus intracellular pools need to be directly examined to assess degradation rather than relying on surface expression as an indicator of the efficiency of this process.

It is also noteworthy that shRNA knockdown of β-COP did not fully reverse Nef-dependent MHC-I and CD4 degradation. This may have resulted from incomplete knockdown of β-COP. However, we also observed a similar phenotype with Nef mutants defective at β-COP binding. Failure to fully reverse degradation may be secondary to a default degradative pathway that exists for all proteins delivered to endosomal pathways. Alternatively, there may be other ways Nef targets these proteins to lysosomes, which have yet to be identified.

Our studies indicate that there are at least three domains needed for Nef to interact efficiently with β-COP. One of these domains (D_123_), is required for dimerization of Nef and is needed to affect a variety of Nef functions [21]. Another region lies within the N-terminal α helical domain of Nef that is specifically required for disruption of MHC-I trafficking and for interactions with AP- 1 [25]. This binding site for β-COP is distinct from that used by AP-1, because recruitment of β-COP does not require Nef's acidic domain or Nef M_20_, whereas AP-1 does [25],[27]. The fact that these Nef mutants bind β-COP, but are still defective at MHC-downmodulation [53] makes sense, because these mutants are also unable to bind the MHC-I cytoplasmic tail [26].

Additional mutants, which focused on the highly conserved stretch of arginines in the N-terminal alpha helical domain of Nef (R_17_XRMRR_22_), revealed that the regions involved in AP-1 and β-COP binding were very closely apposed. However, we determined that mutation of R_17/19_ affected primarily β-COP binding, with only a minimal effect on AP-1 interaction. Thus, these two Nef-interacting proteins have distinct and separable amino acid requirements for binding.

The identification of a β-COP binding domain within a region of Nef that is also required for Nef to accelerate MHC-I degradation confirms the requirement for β-COP in this pathway. In addition, the residual binding of β-COP to these Nef mutants provided suggestive data that another binding site for β-COP existed. Indeed, we were able to confirm prior evidence that a diacidic motif within the C-terminal loop of Nef also promoted an interaction with β-COP and that mutation of this motif reduced CD4 degradation [47]. Finally, we demonstrated that mutation of both the RXR and the diacidic motifs resulted in the greatest defect in β-COP binding. The double mutant did not however result in a greater defect in either MHC-I or CD4 degradation, indicating the role of each motif is distinct and not additive. The discovery of two distinct β-COP binding motifs helps explain why some groups could not confirm the role of the diacidic motif in β-COP binding [48] as both motifs need to be mutated to reliably eliminate an interaction between β-COP and Nef.

There is precedent for such redundancy. For example, there are two AP-1 binding sites within Nef; a dileucine motif within the C-terminal flexible loop [16],[31],[32],[33] as well as a second site that forms upon binding of Nef to the MHC-I cytoplasmic tail. Despite the presence of two AP-1 signals, only one is active in the context of the natural Nef-MHC-I complex [25],[27]. The dileucine motif in the C-terminal flexible loop can become activated to affect MHC-I transport, but only when Nef is artificially fused to the MHC-I cytoplasmic tail [27]. This result indicates there is no inherent inability of this signal to affect MHC-I traffic but rather that something else, such as the structure of the natural complex, causes the dileucine motif to be inactive [27]. The dileucine motif at position 164 is located close to the diacidic motif at position 154 that binds β-COP to promote CD4 degradation. The fact that both of these motifs are inactive when Nef is bound to MHC-I, suggests that much of the C-terminal flexible loop region of Nef is inaccessible under these conditions. Thus, Nef behaves as though it assumes different structural forms in different contexts to differentially expose distinct trafficking signals.

We also present evidence that knock down of β-COP yielded a distinct phenotype from BFA treatment. As described above, BFA is a chemical inhibitor of ARF1, that is known to trigger the reversible collapse of the *cis-medial* Golgi compartments to the ER [57]–[59] by inhibiting an ARF-specific guanine nucleotide-exchange protein (ARF-GEF) [60],[61]. Because ARF1 activity is necessary for recruitment of β-COP to membranes [62], it was possible that the dramatic effects of BFA resulted from the inability for β-COP to function normally. However, our results demonstrating that knockdown of β-COP had no effect on overall Golgi structure indicate that the dramatic effects of BFA are not due solely to disruption of β-COP function in the Golgi.

Given the important role of β-COP in the Golgi, it is surprising that β-COP bound to Nef does not also affect transport of MHC-I through the ER/Golgi. It is possible that our inability to detect an effect of Nef on early transport of MHC-I [25] may be a result of the cell type chosen for these studies. T cells, which are an important natural target of HIV, normally traffic MHC-I through the early secretory pathway slowly [23] and thus it might be difficult to further reduce the trafficking speed through an interaction with β-COP. Interestingly, another group has reported a reduced ER-Golgi exit rate for MHC-I in Nef-expressing HeLa cells [63], which normally transport MHC-I more rapidly than T cells [23]. We have made similar observations in astrocytoma cells expressing higher levels of Nef than typically needed to observe MHC-I downmodulation (Roeth and Collins, unpublished observations). Further studies will be needed to determine whether this effect of Nef plays a role in more physiologically relevant cell systems and whether this effect of Nef might be dependent on β-COP expression.

A recent report indicates that the effect of Nef on internalization of MHC-I, which is only minimally apparent in our system, occurs via a PI3-kinase dependent pathway [64]. This publication reported that CEM cells, which were used in our study, have less PTEN (a phosphatase that inhibits PI3-kinase) than another T cell line used in their study (H9). This deficiency might make it relatively more difficult for us to detect an effect of chemical PI3-kinase inhibitors, but would not affect our ability to detect a PI3-kinase-dependent trafficking pathway. In fact, one would expect the opposite, that the PI3-kinase-dependent pathway would be more active in our system. However, we have found that Nef has a relatively small effect on internalization of MHC-I, and mainly affects MHC-I protein export and degradation. These data have been corroborated in HIV-infected primary T cells [22],[26], which were also found to much lower levels of PTEN than H9 cells did [64].

From a teleological perspective, it makes sense that Nef would have evolved to target early forms of MHC-I, which harbor antigens derived from the newly synthesized viral proteins. Older forms of MHC-I already on the cell surface would be bound to normal cellular antigens and would in fact be protective as they would inhibit killing by natural killer cells that are stimulated to lyse cells with abnormally low MHC-I expression. On the other hand, it makes sense that Nef, an early viral protein, would have evolved to target surface CD4 to rapidly and efficiently remove CD4 in order to prepare the cell for rapid release of viral particles and to render the cell resistant to re-infection. Meanwhile, a late protein, Vpu, is expressed in infected cells and specifically targets the newly synthesized CD4 for degradation, preventing any additional CD4 from reaching the cell surface [65].

In sum, we have found that the HIV Nef protein commandeers the cellular trafficking machinery efficiently by utilizing their natural activities for abnormal purposes. The fact that these pathways may end in a final common step raises the important possibility that inhibitors might be developed that could block multiple Nef functions.

## Materials and Methods

### Cell lines

CEM T cells stably expressing HA-tagged HLA-A2 (CEM HA-HLA-A2) have already been described [25]. Cell lines stably expressing YFP-tagged Rab7 or HA-HLA-A2/CD4 were made by transducing cells with murine retroviral constructs (MSCV YFP-Rab7 or MSCV HA-A2/CD4) as previously described [22], followed by culture in selective media.

### DNA constructs

MSCV YFP-Rab7 was constructed by cloning a filled-in a *Kpn* I-*Xho* I fragment from pEYFP-Rab7 [66] into MSCV puro [67]. MSCV HA-A2/CD4 was constructed using PCR mutagenesis. The first round PCR produced two products: the first utilized 5′ primer (primer 1) 5′-CGGGATCCACCATGCGGGTCACGGCG-3′ and 3′ primer (primer 2) 5′-CTCTGCTTGGCGCCTTCGGTGCCACATCACAGCAGCGACCAC-3′ with MSCV HA-HLA-A2 as the template [25]. The second utilized 5′ primer (primer 3) 5′-GTGGTCGCTGCTGTGATGTGGCACCGAAGGCGCCAAGCAGAG-3′ and 3′ primer (primer 4) 5′-CCTCGAGTCAAATGGGGCTACATGTCTTCTGAAATCGGTGAGGGCACTGG-3′ using CD4 as the template. The second round utilized primers 1 and 4 from the previous PCR reactions plus 1 µl of each purified first round PCR reactions as template. The resulting product was digested with BamHI and XhoI and ligated into MSCV 2.2 [67] digested with BglII and XhoI.

MSCV A2/Nef has been described [26]. MSCV HA-A2/CD4/Nef was constructed using a PCR mutagenesis approach. The first round PCR produced two products: the first utilized 5′ primer (primer 1) 5′-CGGGATCCACCATGCGGGTCACGGCG-3′ and 3′ primer (primer 2) 5′-CCACTTGCCACCCATACTAGTAATGGGGCTACATGT-3′ with MSCV HA-A2/CD4 as the template. The second utilized 5′ primer (primer 3) 5′-ACATGTAGCCCCATTACTATGATGGGTGGCAAGTGG-3′ and 3′ primer (primer 4) 5′- GCGAATTCTCAGCAGTTCTTGAAGTACTC-3′ with NL4-3 Nef open reading frame as template. The second round utilized primers 1 and 4 from the previous PCR reactions plus 1 µl of each purified first round PCR reactions as template. The resulting product was digested with BamHI and EcoRI and ligated into MSCV IRES GFP [68] digested with BglII and EcoRI.

Nef mutants were made by using the PCR mutagenesis approach described previously (Wonderlich et al. 2008). The mutagenesis primers were as follows: R_17/19_A 5′-TGGCCTACTGTAGCGGAAGCAATGAGACGAGCT-3′ and EE_154–155_AA 5′-GTTGAGCCAGATAAGGTAGCAGCGGCCAATAAAGGAGAGA-3′. Each primer, plus its reverse complement were utilized together with additional 5′ and 3′ primers to generate the mutated product. Wild type NL4-3 Nef [MSCV A2/Nef IRES GFP (Roeth et al 2005)] was used as a template for the PCR reaction, except for the double mutant, R_17/19_A/EE_154–155_AA, in which the MSCV R_17/19_A Nef IRES GFP was used as the template. Each mutated PCR product was digested and cloned into MSCV IRES GFP [68] as described previously (Wonderlich et al. 2008).

The FG12 shRNA lentiviral vectors were constructed as previously described [50]. Briefly, complementary primers were annealed together and ligated into vector pRNAi [69] digested with BglII and HindIII. The sequences of the primers were as follows (the target sequence is underlined): shNC (an siRNA directed at GFP, with several base changes [25])- sense 5′-GATCCCCGCTCACACTGAAGTTAATCTTCAAGAGAGATTAACTTCAGTGTGAGCTTTTTGGAAA-3′, antisense 5′-AGCTTTTCCAAAAAGCTCACACTGAAGTTAATCTCTCTTGAAGATTAACTTCAGTGTGAGCGGG-3′, shβ-COP- sense 5′-GATCCCCTGAGAAGGATGCAAGTTGCTTCAAGAGAGCAACTTGCATCCTTCTCATTTTTGGAAA-3′, antisense 5′-AGCTTTTCCAAAAATGAGAAGGATGCAAGTTGCTCTCTTGAAGCAACTTGCATCCTTCTCAGGG-3′; shμ1A- (a mixture of two lentiviruses was used) (1) sense 5′GATCCCCTGAGGTGTTCTTGGACGTCTTCAAGAGAGACGTCCAAGAACACCTCATTTTTGGAAA-3′, antisense 5′-AGCTTTTCCAAAAATGAGGTGTTCTTGGACGTCTCTCTTGAAGACGTCCAAGAACACCTCAGGG-3′, (2) sense 5′- GATCCCCCGACAAGGTCCTCTTTGACTTCAAGAGAGTCAAAGAGGACCTTGTCGTTTTTGGAAA-3′, and antisense 5′- AGCTTTTCCAAAAACGACAAGGTCCTCTTTGACTCTCTTGAAGTCAAAGAGGACCTTGTCGGGG-3′. The pRNAi constructs were digested with XbaI and XhoI to remove the promoter and shRNA sequence. The resulting fragment was ligated into FG12 [50], digested with XbaI and XhoI.

### Virus preparation and transductions

Adenovirus was prepared by the University of Michigan Gene Vector Core facility. Adenoviral and HIV (HXB-EP [6]) transductions of T cells [25] or 373 mg astrocytoma cells [49] have been described previously. Murine retroviral vector (MSCV) expressing Nef was prepared as described previously (Roeth et al. 2005), except that in some cases the retroviral vector supernatants were concentrated by spinning at 14000 RPM for four hours at 4°C. The viral pellet was then resuspended in media to yield a twenty-fold concentrated stock. Lentiviruses expressing shRNA were generated using an approach similar to that already described [50]. Briefly, 293 cells were transfected with the FG12 constructs described above plus pRRE [70], pRSV-Rev [70] and pHCMV-G [71] using Lipofectamine 2000 (Invitrogen). Supernatants from the transfected cells were collected and used to transduce CEM T cells using a spin-transduction protocol.

### Flow cytometry and internalization assays

Intact cells were stained for flow cytometry analysis as previously described [24]. Briefly, HLA-A2 was detected with BB7.2 [72] that had been purified as previously described [22]. Endogenous CD4 was detected using RPA-T4 from Serotec. The secondary antibody was goat anti-mouse-phycoerythrin (BioSource, 1∶250). For experiments using the GFP-expressing FG12 lentivirus for shRNA expression, the GFP-positive cells were gated to identify the subset of transduced cells (generally >90% of cells). Endocytosis assays were performed as previously described with minor modification [22]. Briefly, cells were washed once with Endocytosis Buffer [D-PBS, 10 mM HEPES, 10 µg/ml BSA (NEB)], then stained with primary antibody (described above) for 20 minutes on ice. After washing, the cells were resuspended in RPMI supplemented with 10% fetal bovine serum, 10 mM HEPES buffer, 2 mM L-glutamine, penicillin and streptomycin (R10) (pre-warmed to 37°C) and replicate aliquots were removed and placed on ice for each time point. Cells were then washed and stained with goat anti-mouse-phycoerythrin (BioSource, 1∶250) and the samples were analyzed using a FACScan flow cytometer (Becton Dickinson). Flow cytometry data was processed using FlowJo v4.4.3 software (Treestar Corp.). The mean fluorescence at time zero was set to 100%, and this value was used to calculate the relative surface staining at each subsequent time point.

### Cell surface transport assay

CEM cells transduced with adenoviral vectors as previously described [22] were first incubated in pre-label media [RPMI –Cys –Met (Specialty Media, Inc.)+10% dialyzed FBS (Invitrogen)] for 15 minutes at 37°C. Pulse labeling was performed in pre-label media with 150–200 µCi/ml Pro-mix-L [^35^S] (>1000 Ci/mmol; Amersham Pharmacia) for 30 minutes at 37°C. The cells were then chased in R10 media for 15 minutes at 37°C, followed by two washes with D-PBS. To label the protein that reached the cell surface, the cells were resuspended in D-PBS containing 0.5 mg/ml EZ-Link sulfo-NHS-LC-Biotin (Pierce), and incubated at 37°C for 1 hour. Surface biotinylation was quenched by washing the cells in D-PBS+25 mM Lysine (Fisher).

For Figure 1D, immunoprecipitation of proteins from cell lysates was performed as previously described [25], except that one-third of the total lysate was used for the HLA-A2 immunoprecipitation while two-thirds of the material was used to recover CD4. For immunoprecipitations of ^35^S labeled proteins, 5 µg of BB7.2 and 2.5 µg RPTA4 (BD Pharmingen) were used for HLA-A2 and CD4 respectively. In Figure 1E and 3D, the total cell lysate was immunoprecipitated with anti-HA ascites (HA.11, Covance).

For Figures 1D, 1E and 3D, recovered proteins were released from the beads by boiling in 100 µl of 10% SDS. One third was analyzed directly by SDS-PAGE (Total). The remaining two thirds was brought to a total volume of 1 ml in RIPA Buffer [25], and 40 µl of avidin-agarose (Calbiochem) was added to recover biotinylated proteins. After 2 hours at 4°C, the beads were washed three times with 1 ml RIPA buffer and proteins were separated by SDS-PAGE (Surface).

### Immunofluorescence microscopy

Adeno-transduced CEM cells were adhered to glass slides, fixed, permeabilized, and stained for indirect immunofluorescence as previously described [25]. Bafilomycin treatment was performed as described previously [25]. The following antibodies were utilized to localize proteins via microscopy: Figure 2, and Figures S1 and S4; anti-CD4 (S3.5, Caltag Laboratories) and anti-HLA-A2 (BB7.2); Figure 3; anti-giantin (Covance); Figure 5; anti-CD4 antibody (S3.5, Caltag Laboratories), anti-LAMP-1 (H4A3, BD Pharmingen) and anti-HLA-A2 (BB7.2). Secondary antibodies were obtained from Molecular Probes and were used at a dilution of 1∶250: Giantin, Alexa Fluor 546 goat anti-rabbit; CD4, Alexa Fluor 546 goat anti-mouse IgG2a; LAMP-1, Alexa Fluor 546 goat anti-mouse IgG1; BB7.2 (Figures 2, 5D and S4), Alexa Fluor 647 goat anti-mouse IgG2b; BB7.2 (Figure S1), Alexa Fluor 488 goat anti-mouse IgG2b. See Table S2 for a summary of antibodies used to gather data for Table S1.

For the microscopy based internalization assay in Figure 5A, CEM T cells were allowed to adhere to glass slides, and placed on ice. The cells were washed once with wash buffer (D-PBS, 10 µg/ml BSA (NEB) and 2% goat serum), incubated with anti-CD4 antibody (S3.5, Caltag Laboratories, IF, 1∶25) for 20 minutes, washed once with wash buffer, incubated with Alexa fluor 546 goat anti-mouse IgG2a (Molecular Probes, 1∶250) for 20 minutes and washed once with wash buffer. The zero time point was fixed with 2% paraformaldehyde, while the remaining time points incubated at 37°C for the indicated time. The cells were then fixed with 2% paraformaldehyde. Images were collected using a Zeiss LSM 510 confocal microscope and processed using Adobe Photoshop software. Three-dimensional projections of cells were generated from Z-stacks using Zeiss LSM Image Examiner software. Otherwise, single Z sections through the center of the cell were displayed.

### Electron microscopy

Electron microscopy with CEM cells transduced with adenovirus was performed by the Harvard Medical School (HMS) Electron Microscopy Facility. Frozen samples were sectioned at −120°C, the sections were transferred to formvar-carbon coated copper grids and floated on PBS until the immunogold labeling was carried out. The gold labeling was carried out at room temperature on a piece of parafilm. All antibodies and protein A gold were diluted in 1% BSA. The diluted antibody solution was centrifuged 1 minute at 14,000 rpm prior to labeling to avoid possible aggregates. Grids were floated on drops of 1% BSA for 10 minutes to block for unspecific labeling, transferred to 5 µl drops of primary antibody and incubated for 30 minutes. The grids were then washed in 4 µl drops of PBS for a total of 15 minutes, transferred to 5 µl drops of Protein-A gold for 20 minutes, washed in 4 µl drops of PBS for 15 minutes and 6 µl drops of double distilled water. Contrasting/embedding of the labeled grids was carried out on ice in 0.3% uranyl acetete in 2% methyl cellulose for 10 minutes. Grids were picked up with metal loops (diameter slightly larger than the grid) and the excess liquid was removed by streaking on a filter paper (Whatman #1), leaving a thin coat of methyl cellulose (bluish interference color when dry). The grids were examined in a Tecnai G^2^ Spirit BioTWIN transmission electron microscope and images were recorded with an AMT 2k CCD camera.

### Western blot analyses and immunoprecipitations

For the western blot analysis in Figures 3A, 4C, 7D, 9A, S2, and S3, cells were lysed in PBS 0.3% CHAPS, 0.1% SDS pH 8, 1 mM PMSF, normalized for total protein and separated by SDS-PAGE. Endo H (NEB) digestion was performed according to the manufacturer's protocol. Staining of the western blot was performed using anti-Nef (AG11, [73]) and anti-β-COP (M3A5 [74]), which were purified as previously described [22]. Additional antibodies used were HA (Covance) and μ1 (RY/1 [75]). The secondary antibody for anti-Nef, β-COP, and HA was HRP-rat anti-mouse IgG_1_ (Zymed) and for anti-μ1 was HRP-goat anti-rabbit (Zymed).

For Figure 6B, the IP-western experiment was performed as previously published [26]. Briefly, parental CEM T cells were spin-transduced with murine retroviral supernatant expressing either empty vector, A2/Nef or A2/CD4/Nef. At 72 hours post transduction, the cells were incubated in 20 mM NH_4_Cl for 4 hours. The cells were then treated with DTBP (Pierce) for 40 minutes, quenched per the manufacturer's protocol, and lysed in PBS with 0.3% Chaps and 0.1% SDS. The lysate was pre-cleared and immunoprecipitated with HLA-A2 with BB7.2 chemically crosslinked protein A/G beads (Calbiochem) [25]. The immunoprecipitates were washed in TBS with 0.3% CHAPS and 0.1% SDS. A more stringent IP protocol was used in Figures 6A, 7C, 8E, and S5. For these experiments, CEM cells were transduced with control, wild type Nef, or mutant Nef expressing adenovirus (Figure 6A and 7C) or concentrated MSCV (Figures 8E and S5). At 48 hours post-transduction, the cells were incubated in 20 mM NH_4_Cl for 16 hours. The cells were not crosslinked and were lysed in digitonin lysis buffer (1% digitonin (Wako), 100 mM NaCl, 50 mM Tris pH 7.0, 1 mM CaCl_2_, and 1 mM MgCl_2_). After pre-clear, the lysates were immunoprecipitated with either BB7.2 (Figures 6A and S5) or M3A5 (Figures 7C and 8E) crosslinked to beads. The immunoprecipitates were eluted and analyzed by western blot as described previously [26].

### Pulse-chase analysis of protein degradation

A total of 30 million CEM T cells transduced with wild type or mutant Nef using concentrated MSCV as described above were pulse labeled for 30 minutes with [^35^S]-methionine and cysteine. Half of the cells were collected as the zero time point and stored at −20 degrees. The remaining cells were then chased for 12 hours in RPMI, collected and stored at −20 degrees. Lysates were generated in lysis buffer (PBS 0.3% CHAPS, 0.1% SDS pH 8, 1 mM PMSF) and precleared over night. They were immunoprecipitated for two hours with an anti-HLA-A2 antibody (BB7.2) and washed once in radioimmunoprecipitation assay (RIPA) buffer (50 mM Tris pH 8, 150 mM NaCl, 1% NP-40, 0.5% deoxycholate, 0.1% SDS). The immunoprecipitates were then eluted by boiling in 10% SDS, reprecipitated with an antibody against HA (HA.11, Covance), and washed two times in RIPA buffer. The final immunoprecipitates were then separated by SDS-PAGE, the gel was dried down and analyzed using a phosphorimager.

## Supporting Information

Table S1 Analysis of CD4^+^ structures in Nef-expressing T cells. CEM HLA-A2 cells were transduced with adeno-Nef and analyzed by three-color confocal microscopy as described in Materials and Methods. Discrete CD4^+^ structures were identified and scored for co-localization with HLA-A2 or the indicated organelle marker protein. Data from at least two independent experiments were combined for each protein analyzed.
(0.02 MB DOC)

Table S2 Combinations of antibodies used for immunofluorescence staining for experiments summarized in Table S1.
(0.04 MB DOC)

Figure S1 Bafilomycin treatment increases MHC-I and CD4 co-localization in Nef-expressing cells. CEM HA-HLA-A2 cells were transduced with a control adenovirus (nef^−^) or adeno-Nef (nef^+^) as described in Materials and Methods. At 72 hours later, the cells were treated with bafilomycin or solvent control (DMSO) and stained with antibodies directed against HLA-A2 and CD4 as described in Materials and Methods. Images were taken with a Zeiss confocal microscope and processed with LSM Image Browser and Adobe Photoshop software. Single Z-sections are shown.
(1.44 MB TIF)

Figure S2 A second siRNA directed at β-COP disrupts Nef-dependent MHC-I trafficking. (A) Western blot analysis of protein expression in 373 mg astrocytoma cells transfected with the indicated siRNA. Previously published protocols [25] were used to transfect 373 mg astrocytoma cells with control siRNA (siGFP [25]) an siRNA targeting β-COP (siβ-COP, sense 5′-GGAGAUGUAAAGUCAAAGA-3′, antisense 5′-UCUUUGACUUUACAUCUCC-3′, Ambion) or an siRNA targeting the AP-1 μ subunit (si μ 1 [25]). The data is representative of three experiments. (B) β-COP and μ 1 are required for Nef to efficiently reduce cell surface expression of HLA-A2. HLA-A2 cell surface expression on astrocytoma cells from (A) was assessed by flow cytometry as described in Materials and Methods. The fold downmodulation of HLA-A2 (mean fluorescence intensity of control/mean fluorescence intensity of Nef-expressing cells) for each condition is shown in the upper left corner. (C) Quantitation of HLA-A2 fold downmodulation in Nef expressing cells treated with siRNA. The mean fold downmodulation±standard deviation from three experiments is shown.
(0.24 MB TIF)

Figure S3 (A) Characterization of HA-HLA-A2 protein forms using western blot analysis. CEM T cells expressing HA-HLA-A2 were lysed and treated with either Endo H or neuraminidase. The samples were then analyzed via Western blot. (B) CEM T cells expressing HA-HLA-A2 and Nef or a control adenoviral vector were lysed, normalized for total protein, digested with endo H, and probed for HA-HLA-A2 by Western blotting with an anti-HA antibody.
(0.24 MB TIF)

Figure S4 Shβ-COP does not disrupt co-localization of CD4 and HLA-A2, but does increase the amount of stainable protein within the cell. HLA-A2 CEM cells were transduced with a lentivirus expressing control (shNC) or β-COP (shβ-COP) shRNA. After 3 days, the cells were transduced with adeno-Nef. After three additional days, the cells were stained with antibodies directed against HLA-A2 and CD4 as in Figure S1. Images were taken with an Olympus FV-500 confocal microscope and processed with Adobe Photoshop software. Single Z-sections are shown.
(0.54 MB TIF)

Figure S5 Mutation of R17/19 and E154/155 only slightly diminishes the amount of Nef and AP-1 coprecipitating with HLA-A2. CEM cells expressing HA-HLA-A2 were transduced with a retroviral vector expressing either wild type Nef or the indicated Nef mutant. The cells were immunoprecipitated with an anti-HLA-A2 antibody (BB7.2), and the presence of Nef was assessed by Western blot as described in Materials and Methods. “Control” indicates lysates from parental CEM T cells that lack HLA-A2, but that express wild-type Nef. “Vector only” indicates CEM T cells expressing HA-A2 transduced with empty retroviral vector.
(0.29 MB TIF)
