# Supplementary material for: HIV-1 Nef Targets MHC-I and CD4 for Degradation Via a Final Common β-COP–Dependent Pathway in T Cells
Source: PLoS Pathog. 2008 Aug 22;4(8):e1000131. doi: 10.1371/journal.ppat.1000131 (PMC2515349; doi:10.1371/journal.ppat.1000131)
Supplement: Table S1 — Analysis of CD4+ structures in Nef-expressing T cells. CEM HLA-A2 cells were transduced with adeno-Nef and analyzed by three-color confocal microscopy as described in Materials and Methods. Discrete CD4+ structures were identified and scored for co-localization with HLA-A2 or the indicated organelle marker protein. Data from at least two independent experiments were combined for each protein analyzed. (0.02 MB DOC) [file ppat.1000131.s001.doc]

| **Marker** | **Organelle** | **Number of colocalizing vesicles** | **Total Number of CD4 positive vesicles** | **% CD4**  **Colocalization** |
| --- | --- | --- | --- | --- |
| **HLA-A2** | **N/A** | **306** | **333** | **91.9** |
| **γ-adaptin** | **TGN/Endosomes** | **29** | **78** | **37.2** |
| **EEA1** | **Early Endosomes** | **48** | **125** | **38.4** |
| **YFP-Rab7** | **Late Endosomes** | **49** | **52** | **94.2** |
| **Lamp1** | **Lysosomes** | **92^1^** | **130** | **70.8** |

**^1^The majority of CD4 co-localized with weakly Lamp1-positive structures.**
