# Supplementary material for: HIV-1 Nef Targets MHC-I and CD4 for Degradation Via a Final Common β-COP–Dependent Pathway in T Cells
Source: PLoS Pathog. 2008 Aug 22;4(8):e1000131. doi: 10.1371/journal.ppat.1000131 (PMC2515349; doi:10.1371/journal.ppat.1000131)
Supplement: Table S2 — Combinations of antibodies used for immunofluorescence staining for experiments summarized in Table S1. (0.04 MB DOC) [file ppat.1000131.s002.doc]

| **Molecule**  **Detected** | **Primary Ab** | **Secondary Ab** |
| --- | --- | --- |
| **HLA-A2** | **BB7.2**  **(20 μg/ml)** | **GAM-IgG2b**  **AlexaFluor 647**  **(1:250, Molecular Probes)** |
| **CD4** | **RPAT4**  **(1:50, BD Pharmingen)** | **GAM-IgG1**  **AlexaFluor 546**  **(1:250, Molecular Probes)** |
| **YFP-Rab7** | NA | **NA** |
| **HLA-A2** | **BB7.2**  **(20 μg/ml)** | **GAM-IgG2b**  **AlexaFluor 488**  **(1:250, Molecular Probes)** |
| **CD4** | **α-CD4-FITC**  **(1:25, Caltag)** | **NA** |
| **γ-adaptin** | **Clone 88**  **(1:25, BD Pharmingen)** | **GAM-IgG1**  **AlexaFluor 546**  **(1:250, Molecular Probes)** |
| **EEA1** | **Clone 14**  **(1:200, BD Pharmingen)** | **GAM-IgG1**  **AlexaFluor 546**  **(1:250, Molecular Probes)** |
| **Lamp1** | **H4A3**  **(1:500, BD Pharmingen)** | **GAM-IgG1**  **AlexaFluor 546**  **(1:250, Molecular Probes)** |
