# Supplementary figures and images for: HIV-1 Nef Targets MHC-I and CD4 for Degradation Via a Final Common β-COP–Dependent Pathway in T Cells
Source: PLoS Pathog. 2008 Aug 22;4(8):e1000131. doi: 10.1371/journal.ppat.1000131 (PMC2515349; doi:10.1371/journal.ppat.1000131)

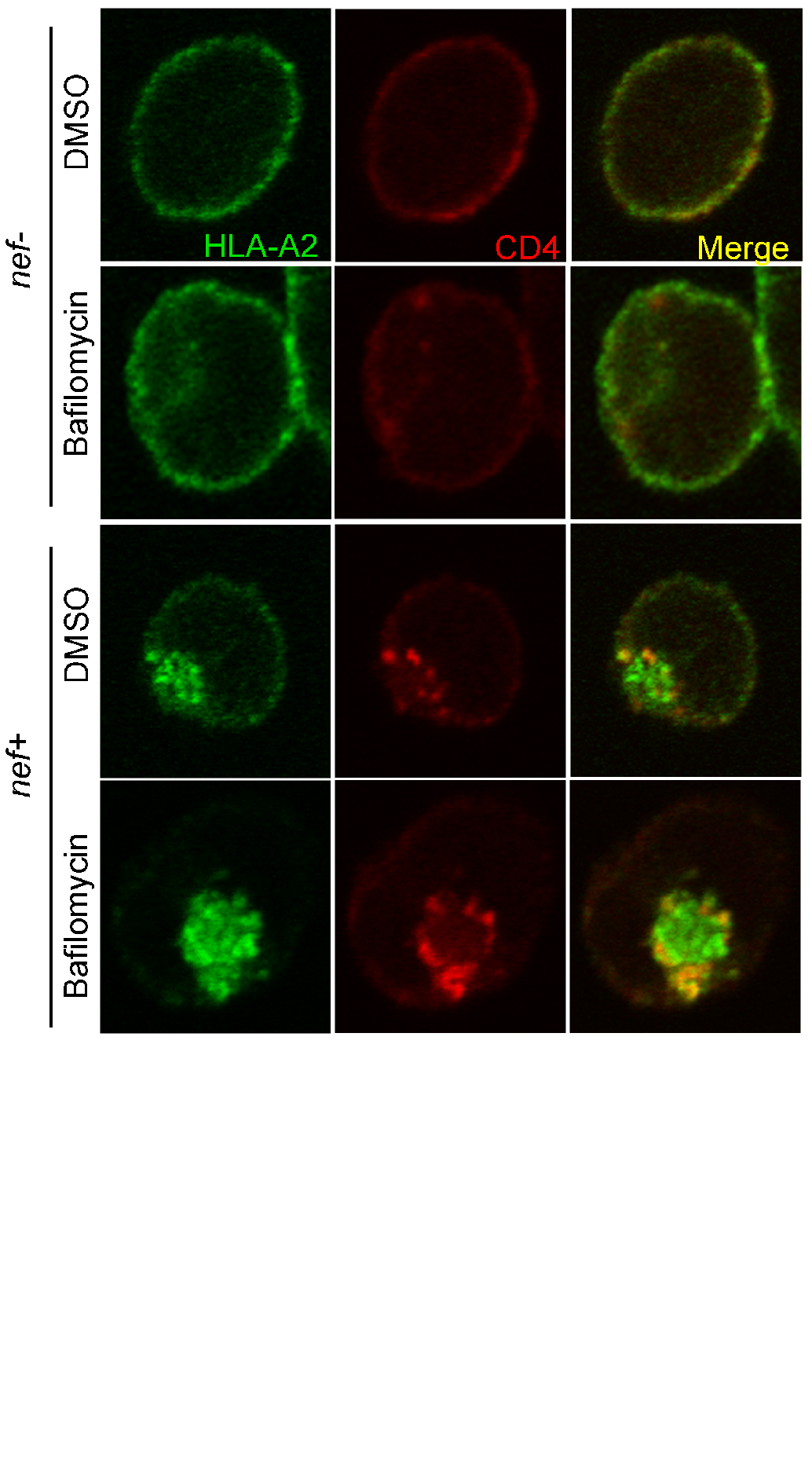

Supplement: Figure S1 — Bafilomycin treatment increases MHC-I and CD4 co-localization in Nef-expressing cells. CEM HA-HLA-A2 cells were transduced with a control adenovirus (nef−) or adeno-Nef (nef+) as described in Materials and Methods. At 72 hours later, the cells were treated with bafilomycin or solvent control (DMSO) and stained with antibodies directed against HLA-A2 and CD4 as described in Materials and Methods. Images were taken with a Zeiss confocal microscope and processed with LSM Image Browser and Adobe Photoshop software. Single Z-sections are shown. (1.44 MB TIF) [file ppat.1000131.s003.tif]

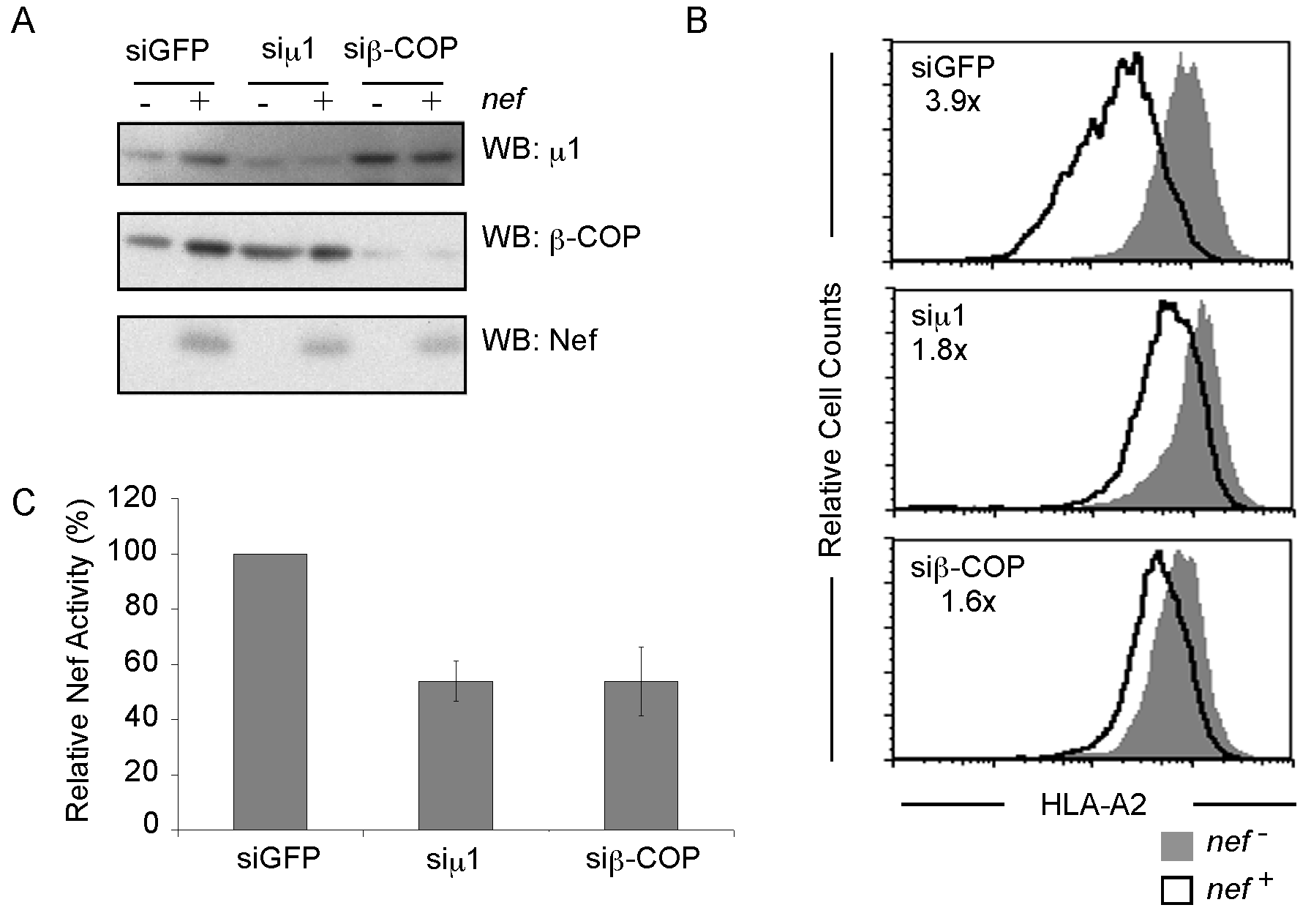

Supplement: Figure S2 — A second siRNA directed at β-COP disrupts Nef-dependent MHC-I trafficking. (A) Western blot analysis of protein expression in 373 mg astrocytoma cells transfected with the indicated siRNA. Previously published protocols [25] were used to transfect 373 mg astrocytoma cells with control siRNA (siGFP [25]) an siRNA targeting β-COP (siβ-COP, sense 5′-GGAGAUGUAAAGUCAAAGA-3′, antisense 5′-UCUUUGACUUUACAUCUCC-3′, Ambion) or an siRNA targeting the AP-1 μ subunit (si μ 1 [25]). The data is representative of three experiments. (B) β-COP and μ 1 are required for Nef to efficiently reduce cell surface expression of HLA-A2. HLA-A2 cell surface expression on astrocytoma cells from (A) was assessed by flow cytometry as described in Materials and Methods. The fold downmodulation of HLA-A2 (mean fluorescence intensity of control/mean fluorescence intensity of Nef-expressing cells) for each condition is shown in the upper left corner. (C) Quantitation of HLA-A2 fold downmodulation in Nef expressing cells treated with siRNA. The mean fold downmodulation±standard deviation from three experiments is shown. (0.24 MB TIF) [file ppat.1000131.s004.tif]

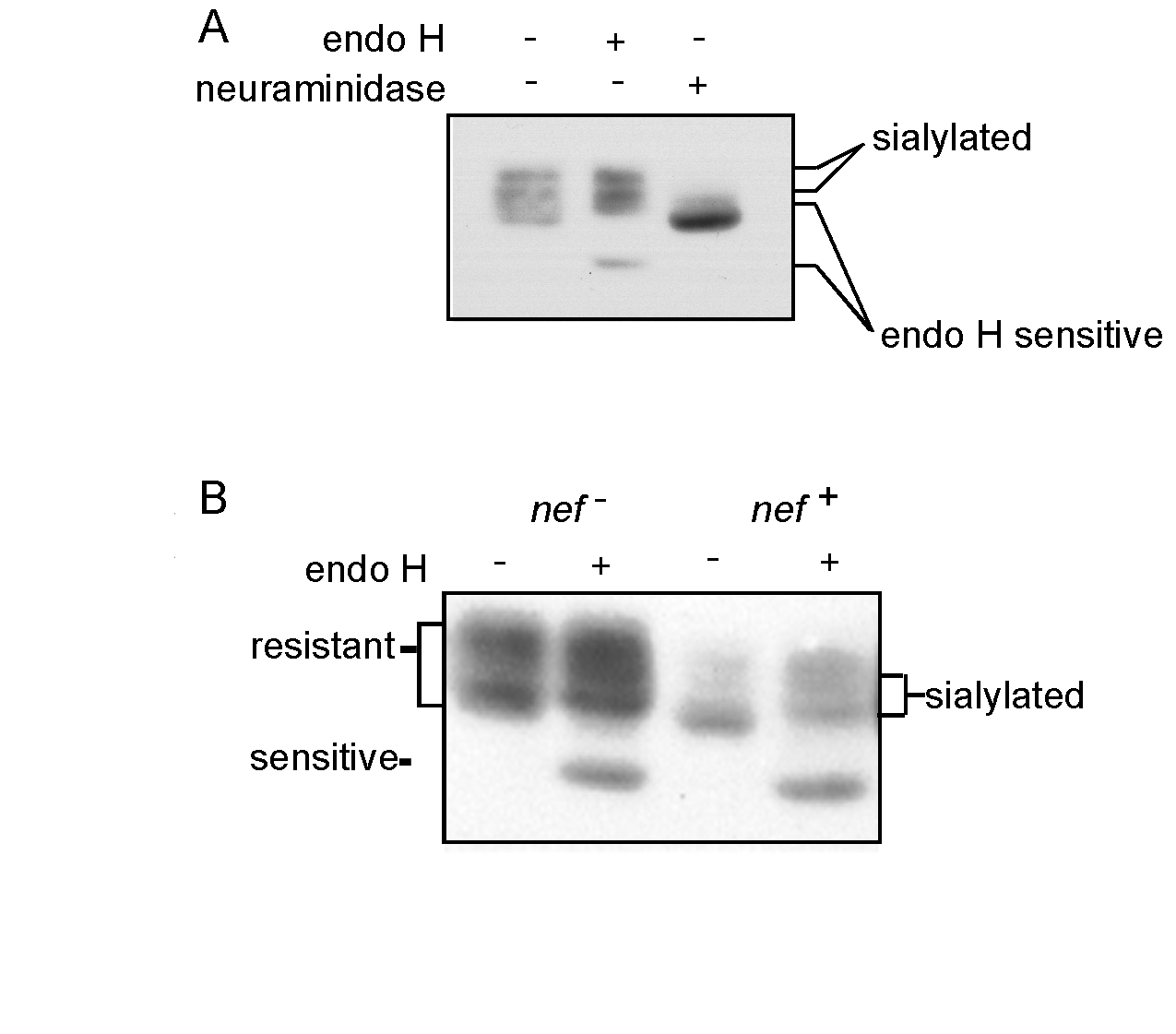

Supplement: Figure S3 — (A) Characterization of HA-HLA-A2 protein forms using western blot analysis. CEM T cells expressing HA-HLA-A2 were lysed and treated with either Endo H or neuraminidase. The samples were then analyzed via Western blot. (B) CEM T cells expressing HA-HLA-A2 and Nef or a control adenoviral vector were lysed, normalized for total protein, digested with endo H, and probed for HA-HLA-A2 by Western blotting with an anti-HA antibody. (0.24 MB TIF) [file ppat.1000131.s005.tif]

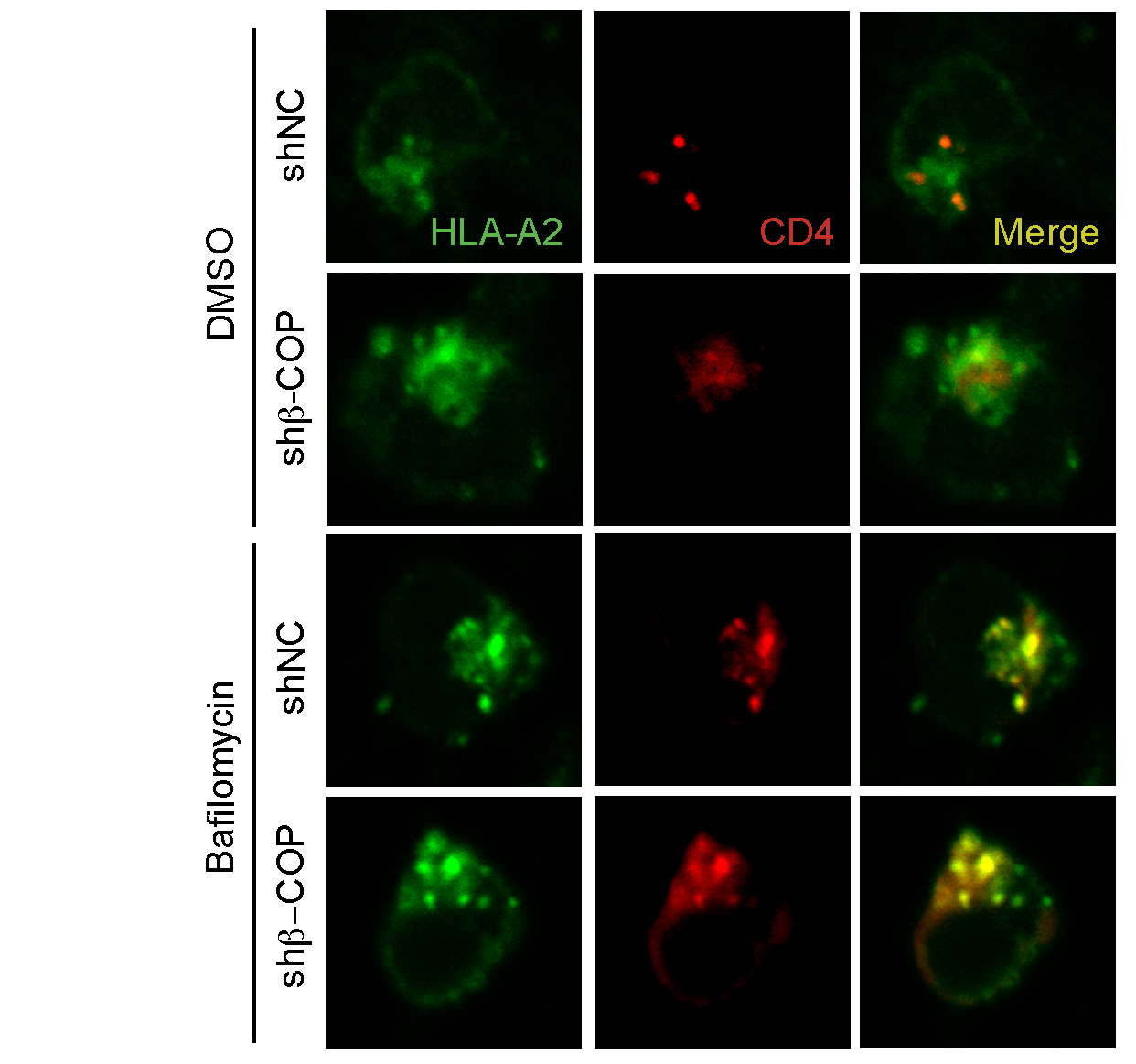

Supplement: Figure S4 — Shβ-COP does not disrupt co-localization of CD4 and HLA-A2, but does increase the amount of stainable protein within the cell. HLA-A2 CEM cells were transduced with a lentivirus expressing control (shNC) or β-COP (shβ-COP) shRNA. After 3 days, the cells were transduced with adeno-Nef. After three additional days, the cells were stained with antibodies directed against HLA-A2 and CD4 as in Figure S1. Images were taken with an Olympus FV-500 confocal microscope and processed with Adobe Photoshop software. Single Z-sections are shown. (0.54 MB TIF) [file ppat.1000131.s006.tif]

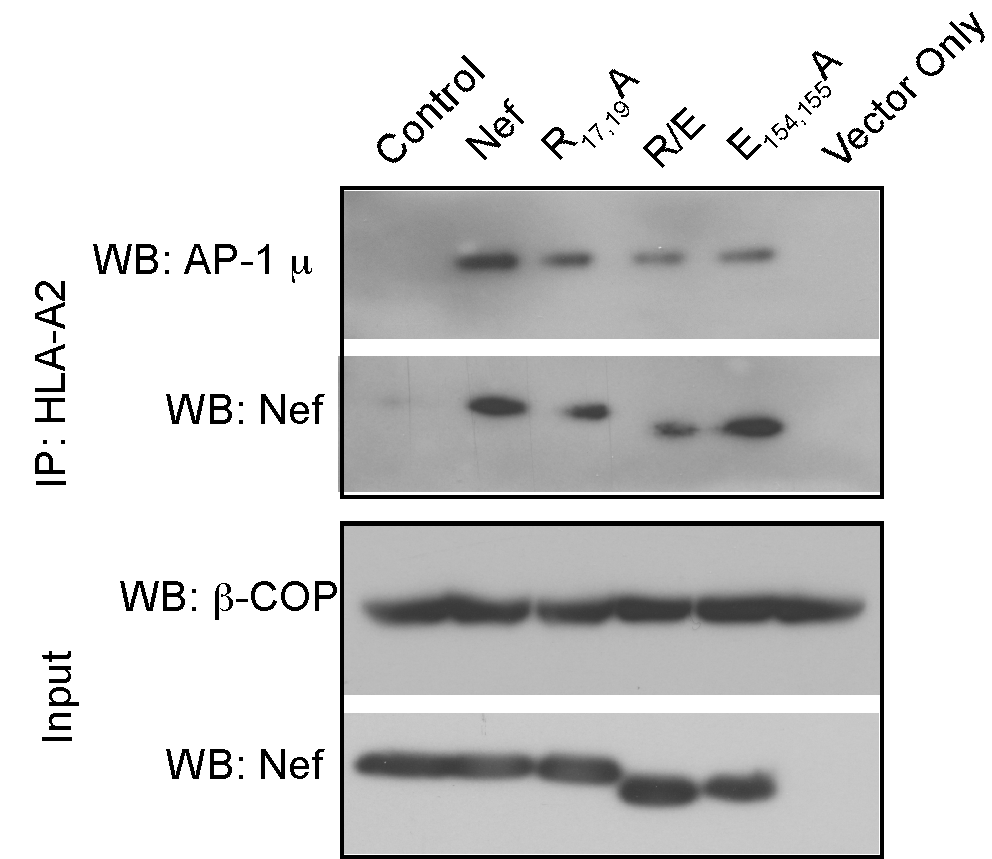

Supplement: Figure S5 — Mutation of R17/19 and E154/155 only slightly diminishes the amount of Nef and AP-1 coprecipitating with HLA-A2. CEM cells expressing HA-HLA-A2 were transduced with a retroviral vector expressing either wild type Nef or the indicated Nef mutant. The cells were immunoprecipitated with an anti-HLA-A2 antibody (BB7.2), and the presence of Nef was assessed by Western blot as described in Materials and Methods. “Control” indicates lysates from parental CEM T cells that lack HLA-A2, but that express wild-type Nef. “Vector only” indicates CEM T cells expressing HA-A2 transduced with empty retroviral vector. (0.29 MB TIF) [file ppat.1000131.s007.tif]
